# Genome editing outcomes reveal mycobacterial NucS participates in a short-patch repair of DNA mismatches

**DOI:** 10.1093/nar/gkae402

**Published:** 2024-05-15

**Authors:** Tanjina Islam, Eric A Josephs

**Affiliations:** Department of Nanoscience, University of North Carolina at Greensboro, Greensboro, NC 27401, USA; Department of Nanoscience, University of North Carolina at Greensboro, Greensboro, NC 27401, USA; Department of Biology, University of North Carolina at Greensboro, Greensboro, NC 27401, USA

## Abstract

In the canonical DNA mismatch repair (MMR) mechanism in bacteria, if a nucleotide is incorrectly mis-paired with the template strand during replication, the resulting repair of this mis-pair can result in the degradation and re-synthesis of hundreds or thousands of nucleotides on the newly-replicated strand (long-patch repair). While mycobacteria, which include important pathogens such as *Mycobacterium tuberculosis*, lack the otherwise highly-conserved enzymes required for the canonical MMR reaction, it was found that disruption of a mycobacterial mismatch-sensitive endonuclease NucS results in a hyper-mutative phenotype, leading to the idea that NucS might be involved in a cryptic, independently-evolved DNA MMR mechanism, perhaps mediated by homologous recombination (HR) with a sister chromatid. Using oligonucleotide recombination, which allows us to introduce mismatches specifically into the genomes of a model for *M. tuberculosis, Mycobacterium smegmatis*, we find that NucS participates in a direct repair of DNA mismatches where the patch of excised nucleotides is largely confined to within ∼5–6 bp of the mis-paired nucleotides, which is inconsistent with mechanistic models of canonical mycobacterial HR or other double-strand break (DSB) repair reactions. The results presented provide evidence of a novel NucS-associated mycobacterial MMR mechanism occurring *in vivo* to regulate genetic mutations in mycobacteria.

## Graphical Abstract

**Graphical Abstract:**
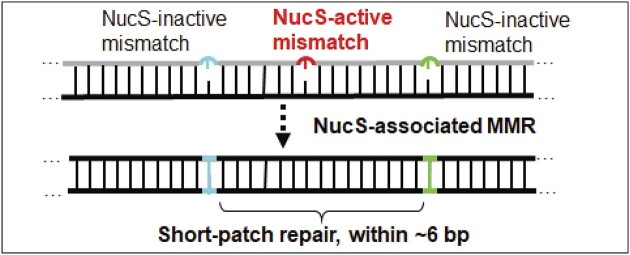

## Introduction

In 2021, there were 10 million incident cases of tuberculosis (TB) worldwide, about 3.9% (450 000) of which were resistant to first line drug rifampicin or multiple antibiotics; this figure represents about 18% of all previously-treated cases (1). *Mycobacterium tuberculosis*, the pathogenic bacteria that causes TB, acquires drug resistance exclusively through chromosomal mutation events, especially single-nucleotide polymorphisms (SNPs) (2,3). However, there is currently an incomplete understanding of the molecular processes that govern the mechanisms of mutation and mutation avoidance in mycobacteria. In nearly all other organisms, except for some archaea and actinobacteria which includes mycobacteria, the rate of genetic mutation and in particular of transition mutations that often occur during replication are tightly controlled by a MutS/MutL-coordinated DNA mismatch repair (MMR) mechanism (4). Immediately after replication, the MMR reaction corrects any incorrectly incorporated or ‘mismatched’ nucleotides that would become permanent genetic mutations if left unrepaired. For example, in the well-studied DNA MMR reaction in *Escherichia coli*, after MutS recognizes a mismatched nucleotide in double-stranded DNA, MutS together with MutL activates latent nicking endonuclease MutH, which then nicks hemi-methylated DNA at d(GATC) sites on the unmethylated strand—an epigenetic signal that discriminates the newly-replicated from the template strand (5). Helicases and exonucleases are loaded at the nicked site and degrade the newly-replicated strand through the mismatch so the strand can be re-synthesized along that tract. Strand degradation and resynthesis can be coordinated between the epigenetic signals and the mismatch even if they are separated by hundreds or thousands of nucleotides in what is known as ‘long-patch’ repair. During senescence, MMR proteins also contribute to the recognition and repair of chemically damaged nucleotides and inhibit improper recombination events between divergent sequences (6–8).

In cells with inactivated MMR pathways, the basal mutation rate is increased 100-fold relative to cells with active MMR (5). However, most actinobacteria have similar basal mutation rates as bacteria with MutS/MutL-coordinated MMR pathways, despite lacking any known homologues of MMR proteins MutS or MutL (9–11). This was originally thought to be a result of unusually high-fidelity mycobacterial DNA polymerases (12). Rather, it was recently discovered (13) that disruption of the mycobacterial gene *nucS*, which encodes a mismatch-sensitive endonuclease, resulted in the same hyper-mutative and hyper-recombinative phenotypes observed for other bacteria in which *mutS* or *mutL* had been disrupted. The types of mutations in the *ΔnucS* strains were primarily transitions, which commonly occur during replication when mispairing by DNA polymerase occurs and whose increased prevalence is are a hallmark of the mutational spectrum of MMR-deficient cells. These findings led to the hypothesis that NucS might be an involved in an evolutionarily-divergent DNA MMR mechanism. *In vitro*, actinobacterial NucS enzymes recognize double-stranded DNA containing mis-paired dG-dG, dG-dT and dT-dT nucleotides and nicks the DNA two nucleotides 5′- of the mismatch on both strands, and endonuclease activity requires association with the beta clamp of the replisome (14,15). However, not much is known about the molecular mechanisms by which NucS activity results in the suppression of a hyper-mutative phenotype. It has been proposed mismatched nucleotides could be corrected during repair of that double-strand breaks (DSBs) caused by NucS by homologous recombination (HR) with a sister chromatid through gene conversion (16). NucS is evolutionarily related to an archaeal mismatch-sensitive endonuclease EndoMS which has also been bioinformatically associated with the enzymes responsible for HR in archaea (17). Alternatively, in principle, a mismatch-sensitive endonuclease might simply induce cell death in members of the bacterial population that are hyper-mutative for other reasons, rather than participating in the correction and repair of the mismatched nucleotides itself.

**Figure 1.**
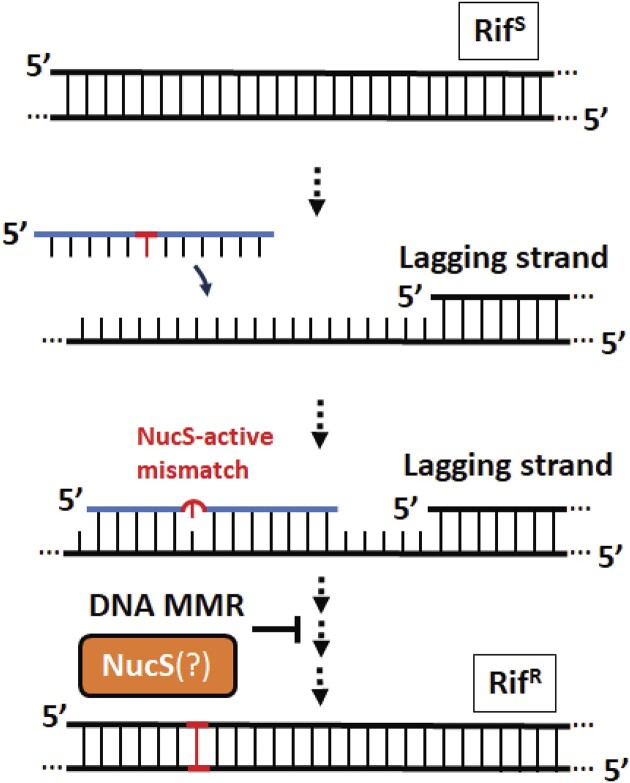
A schematic model oligonucleotide recombination (OR), which is attenuated by DNA mismatch repair (MMR) processes, to probe the involvement of NucS in the repair of mismatched nucleotides in M. smegmatis introduced during OR. During OR, short oligonucleotides are introduced into cells and base-pair with the lagging-strand template, and if there are any mismatched nucleotides on the oligonucleotide they become permanent genetic mutations if left unrepaired before a subsequent round of replication. Mismatches left unrepaired after introduction into the rpoB gene by OR results in a transition from a rifampicin-sensitive (Rif^S^) phenotype to a rifampicin resistant (Rif^R^) phenotype.

Typically, DNA MMR activity and hypermutation are characterized using spontaneous antibiotic resistance assays that can provide an estimate of mutation rates under certain conditions by observing the frequency at which a microorganism acquires one of a spectrum of mutations that result in resistance to certain antibiotics, such as mutations in the gene for the beta subunit of RNA polymerase *rpoB* that result in resistance to rifampicin (13). Mutation accumulation studies using whole genome sequencing have also been performed to understand the role NucS plays in genome maintenance and mutation, which confirmed the role of NucS in limiting transition mutations in *Mycobacterium smegmatis*, a non-pathogenic model of *M. tuberculosis*, that would be caused by dG–dG, dG–dT and dT–dT mis-pairing during replication (18). Using a genome engineering technique known as oligonucleotide recombination (OR) (Figure 1) (19–22), where engineered oligonucleotides that contain mismatched nucleotides with the lagging-strand template are introduced into bacteria and incorporated into the genome during replication, we are able to directly evaluate different molecular mechanisms associated with NucS-associated mutation avoidance in *M. smegmatis*. We find that genomic mismatches introduced during OR are rejected in a manner consistent with the biochemical association of NucS with various combinations of mismatched nucleotides (14,15), but that these mismatches can be repaired in a way that is inconsistent with both the long-patch repair of canonical MMR and canonical mycobacterial DSB repair mechanisms like HR or non-homologous end-joining (NHEJ) (23,24). Our results show that a mycobacterial MMR can occur involving NucS within a short patch (less than about ∼6–9 nt) of the mis-paired nucleotides. We also find that that compound mismatches (25) introduced *via* OR can help to evade NucS-mediated repair and allow for genome editing of mycobacterial species in a manner that we expect will prove useful for probing mycobacterial biology and virulence. The results presented here provide direct evidence that NucS can participate in a mycobacterial MMR reaction that from the outcomes of OR gene editing appears to be mechanistically distinct from repair by HR or other canonical mycobacterial DSB repair mechanisms.

## Materials and methods

### Preparation of *M. smegmatis* strains

*M. smegmatis* strain mc^2^155 (American Type Culture Collection) and *nucS*-knockout strain (prepared using ORBIT (26), a gift from Kenan Murphy of University of Massachusetts Chan Medical School) were prepared following the protocol of Ref. (27). Briefly, these strains were cultured in 7H9 broth (Millipore Sigma) containing Middlebrook ADC Growth Supplement, carbenicillin (50 ug/ml), cycloheximide (10 ug/ml), and 0.25% Tween 80 at 37°C until saturated (∼2–3 days). After 3 days of culture when the cells have reached OD_600_ 0.8–1.0, 5 ml cultures were re-diluted into 50 ml 7H9 broth and transferred into large culture flask to grow overnight. Cells were pelleted by centrifuging at 3600g for 10 min at 4°C and the supernatant discarded, then the cells were washed with 1/2 vol (25 ml glycerol for 50 ml cell culture) 10% sterile glycerol by pipetting to disperse the cells. Washing the cells were performed in similar pattern with 1/4th, 1/8th, 1/10th volume of 10% sterile glycerol by centrifuging at 3600g for 10 min at 4°C with discarding the supernatant. The cells were resuspended in 1/25 vol (2 ml) 10% sterile glycerol to aliquot in 100 μl of cells in microcentrifuge tubes, and stored at −80°C for further experiment.

Electrocompetent cells were incubated for 10 min on ice, mixed with 50 ng of plasmid pJV62 (28) (pJV62 was a gift from Graham Hatfull (Addgene plasmid # 26 910; http://n2t.net/addgene:26910; RRID:Addgene_26 910)), and transferred to chilled 0.2 mm cuvette then electroporated using a BioRad pulse-controller (2.5 kV, 1000 Ω, 25 uF). Plasmid pJV62 expresses the mycobacteriophage Che9c gene 61, a single-stranded DNA recombinase that significantly increases the efficiency of oligonucleotide recombination in mycobacteria, under an inducible acetamidase promoter. The transformants were recovered in 7H9 broth then placed in a shaking incubator at 37°C shaker overnight. The cells were plated on 7H10 agar (Millipore Sigma) plates containing 50 ug/ml kanamycin and incubated at 37°C for 3–5 days until visible colonies appeared.

### Oligonucleotide recombination

All experiments were performed in a manner similar to as described in (28) and in three replicates. 50 ml solutions of wild-type and *nucS* knock-out strains containing pJV62 plasmid were cultured in 7H9 broth with kanamycin for 3 days after which 2% (mass) acetamide to induce the expression of Che9c gene 61, and then allowed to grow for 24 h. Bacteria were then made electrocompetent again following the above protocol prior to electroporation with 100 ng of oligonucleotides (either individually or pooled as described in the main text). The sequences of all oligonucleotides are listed in Supplementary Table S1.

After transformation, the cultures were recovered overnight in 10 ml of 7H9 media and plated in 7H10 agar plate supplemented with 50 ug/ml rifampicin and incubated at 37°C until visible colonies appeared. For sequencing, instead of plating after the recovery step, 50 ug/ml rifampicin was added to each overnight culture directly, then incubated with shaking at 37°C.

After 3 days, 10 ml of cell cultures were transferred into tube to centrifuge at 3600 rpm for 15 min and the supernatant discarded. Cells were resuspended in 1 ml glucose-tris-EDTA (GTE) solution (25 mM Tris–HCl, pH 8.0, 10 mM EDTA, 50 mM glucose) then centrifuged for 10 min and the supernatant discarded. Cells were resuspended in 450 ul GTE solution with 50 ul of a 10 mg/ml lysozyme in 25 mM Tris–HCl and incubated at 37°C overnight. 100 ul 10% sodium dodecyl sulfate and 50 ul 10 mg/ml proteinase K were added to the cells and incubated at 55°C for 40 min, followed by the addition of 200 ul 5 M NaC, a gentle mixing, and the addition of 160 uL of a cetyltrimethylammonium bromide (CTAB) (10 g CTAB and 4.1 g NaCl in 90 ml dH_2_O.) was added and incubated at 65°C for 10 min. After 10 min, cells were washed with 1 ml chloroform:isoamyl alcohol (24:1) and centrifuged at 3600 rpm for 10 min. The supernatant was discarded and the sample washed again. The supernatant was discarded and 560 ul (0.7× by vol) isopropanol was added and mixed gently by inversion until the DNA has precipitated out of solution. This solution was incubated at room temperature for 5 min and centrifuged for 10 min. After discarding the supernatant, cells were washed with 1 ml 70% ethanol and centrifuged for 10 min. The supernatant was discarded and air-dried pellet for 15 min. The pellets were resuspended in 50 μl TE buffer and incubate at 37°C to dissolve pellet and concentrations were measured in Qubit fluorometer (Thermo Fisher). These extracted genomic DNA were stored at −20°C for further analysis

### Sequencing and analysis

For Sanger sequencing, PCR using the purified genomic DNA was performed with primers 5′-ACCGAAAAGGGCACCTTCAT-3′ and 5′-ACCGATCAGACCGATGTTGG-3′ to amplify the region of the *rpoB* gene of interest using OneTaq 2X Master Mix with Standard Buffer (NEB). Sanger sequencing was performed by Azenta Life Sciences (South Plainfield, NJ).

For next generation sequencing (NGS), PCR was performed with genomic DNA with primers containing partial illumina adapter sequences: 5′-ACACTCTTTCCCTACACGACGCTCTTCCGATCTCCGCAGACCCTGATCAACAT-3′ and 5′-GACTGGAGTTCAGACGTGTGCTCTTCCGATCTATCAGACCGATGTTGGGACC-3′. Sequencing was performed by Azenta Life Sciences using their Amplicon E-Z services, which guarantees >50 000 reads per sequencing run.

The lengths of the sequencing reads were measured using code written in-house for MATLAB (MathWorks; Natick, MA) by checking for the sequences immediately flanking those complementary to the oligonucleotides, 5′-GCCGGCGCGCTCACGGGACA-3′ and 5′-AACTGCGACAGCTGGCTGGT-3′. After confirming that nearly all of the repair products were full length (lacking indels), we determined mutation rates for all sequencing reads containing (i) the flanking sequences listed immediately above and (ii) the *rpoB* c.1327A > G mutation indicative of successful oligonucleotide incorporation by searching the sequencing using a regular expression (regexp) for the oligonucleotide sequence with wildcards ([A,T,C,G]) at sites where pooled oligonucleotides had differences from the wild-type sequence. The number of reads at the wildcard locations that differed from the wild-type sequence (mutations) were normalized to the number of reads with containing the *rpoB* c.1327A > G mutation. Reads and mutations were only considered valid if the forward and reverse reads across the entire oligonucleotide sequence were identical.

## Results

During OR (22,29,30), short oligonucleotides are introduced into cells and base-pair with the lagging-strand template, and if there are any mismatched nucleotides on the oligonucleotide they become permanent genetic mutations if left unrepaired before a subsequent round of replication (Figure 1A) (19–21). While genome engineering by OR has been demonstrated in *M. tuberculosis* and *M. smegmatis* (27,28,31,32), OR is not commonly used in mycobacteria, perhaps because of an incomplete understanding of mismatch repair processes, which can attenuate OR efficiencies, in mycobacteria (33). There is significant evidence that OR occurs during replication in mycobacteria and other bacteria, as there is a strong bias in OR efficiency favouring oligonucleotides that base-pair with the lagging-strand template *vs*. the leading-strand template (28), and OR does not occur when the complementary DNA is not actively replicating (20). A re-evaluation of previously-reported OR efficiencies in mycobacteria in light of the discovery of NucS and its biochemical characterization that it cleaves dG–dG, dG–dT and dT–dT mismatches in double-stranded DNA suggested that OR might be attenuated by the activity of NucS (28).

**Figure 2.**
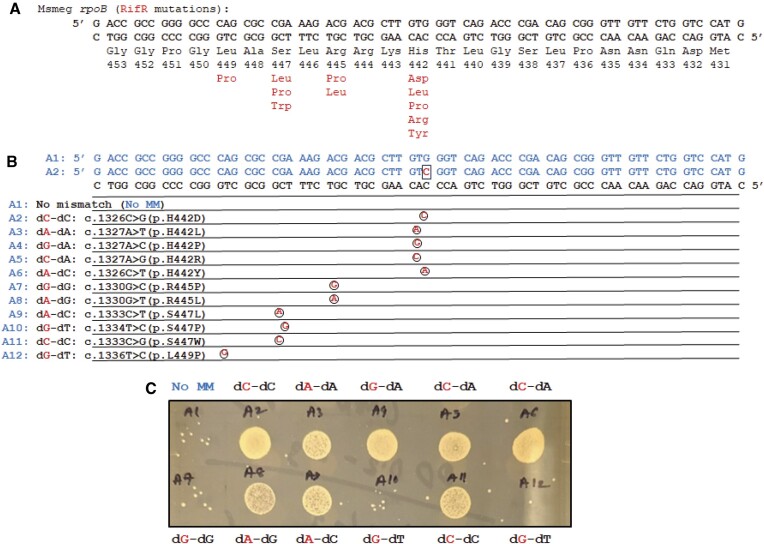
Gene editing-generated rifampicin resistance reveals the types of DNA mismatches rejected during OR. (**A**) Lagging strand sequence (top strand) and its complement (bottom strand) with codons and numbering for M. smegmatis rpoB. Several (red) mutations in the rpoB gene are known to result in resistance to first-line antibiotic rifampicin (Rif^R^ phenotype). (**B**) 12 oligonucleotides (blue) differ by a single nucleotide to introduce different varieties of mismatched nucleotides that produce known Rif^R^ mutations. Only the sequence differences with the control oligonucleotide (oligo A1), which base-pairs perfectly with the lagging-strand template are shown for oligos A2–A12. (**C**) *M. smegmatis* with Rif^R^ phenotype are efficiently generated when using oligonucleotides that introduce dC–dC, dA–dA, dG–dA, dC–dA, dA–dC and dA–dG mismatches, but not dG–dT or dG–dG mismatches, known targets of NucS.

Because OR has been used to probe cellular processes in *E. coli*, yeast, and human cells (25,34–39)—in particular, DNA MMR processes (40,41)—we sought to determine whether oligonucleotides with mismatches introduced during OR into *M. smegmatis* were rejected by NucS. From mutation accumulation studies involving NucS-knockout strains of *M. smegmatis*, it has been determined the NucS can influence the emergence of antibiotic resistance to first-line antibiotic rifampicin that arise from a spectrum of mutations in gene for the beta subunit of RNA polymerase *rpoB* (Figure 2A) (42,43). Therefore, we designed a series of 11 nearly-identical oligonucleotides that contained single mis-pairing nucleotides across 6 codons in a region of *M. smegmatis* gene *rpoB* that would generate mutations that are known to result in resistance to rifampicin if the mismatched nucleotides are left unrepaired (Figure 2B); failure to survive in rifampicin would reveal which mismatches are rejected *in vivo* during OR (Figure 1).

Cultures of log-phase *M. smegmatis* were individually electroporated with these eleven oligonucleotides and a control oligonucleotide containing no mis-pairing nucleotides (and therefore designed to cause no mutation or rifampicin resistance), allowed to recover for 3 days, then plated on solid media containing rifampicin (28). *M. smegmatis* have a doubling time of ∼7 h, and this allows mismatched nucleotides to resolve into permanent genetic mutations after replication. There was a dramatic enrichment in the population of survivors compared to the control when the cultures were transfected with oligonucleotides that introduced dC–dC, dA–dA, dG–dA, dA–dG, dC–dA and dA–dC mismatches (Figures 2C and S1); each of those mismatched nucleotide pairs are not recognized by NucS *in vitro* (14,15). That enrichment in the number of rifampicin-resistant survivors, which was observed to occur in approximately 10–25% of those total populations (Supplementary Figure S2), was not observed in oligonucleotides that introduced dG–dG or dG–dT mismatches that, *in vitro*, are cleaved by NucS. This provided initial evidence that, as expected, mismatches introduced using OR are rejected through a NucS-mediated mechanism, which was further supported by additional evidence provided below.

**Figure 3.**
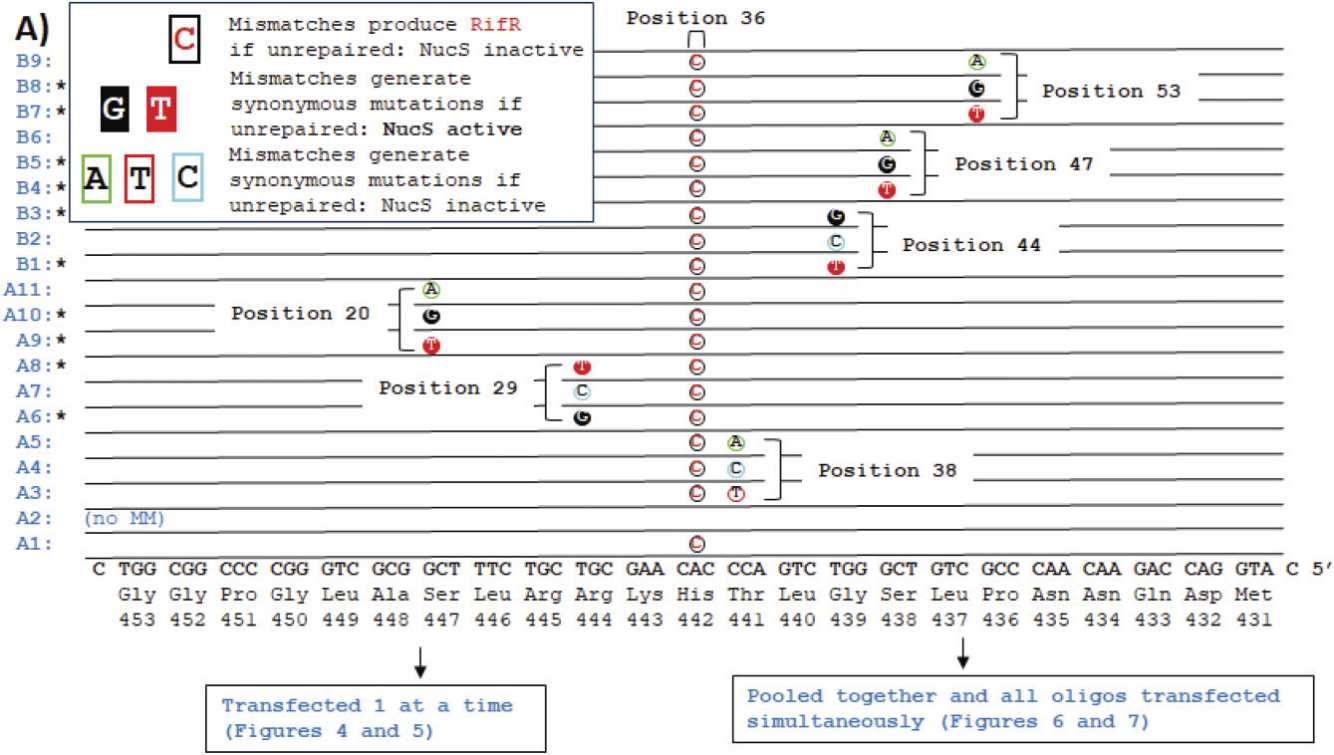
Oligonucleotides to introduce NucS-active or NucS-inactive mis-pairs near a mis-pair to generate a rifampicin-resistance mutation. Oligonucleotides (blue) where NucS-active (filled in circles) and NucS-inactive (open circles) mis-pairs are introduced to generate synonymous mutations in rpoB along with mis-pairs to generate rifampicin resistance (open circles with red letters). Below, lagging strand template sequence. Oligonucleotides were transfected into *M. smegmatis* cultures individually for experimental results described in Figures 4 and 5, and also pooled together and transfected simultaneously into *M. smegmatis* cultures for the experimental results described in Figures 6 and 7. Positions labelled relative to oligonucleotide 5′.

**Figure 4.**
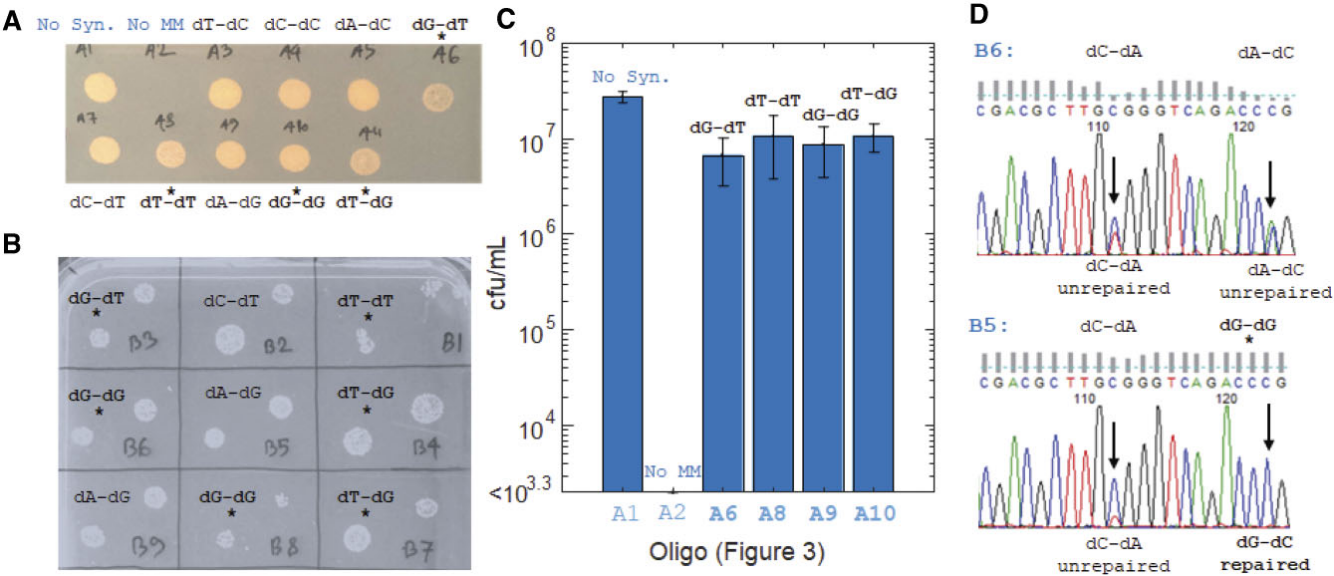
Influence of nearby NucS-active and NucS-inactive mis-pairs on the ability to introduce a NucS-inactive mispair that generates a Rif^R^ phenotype. (**A, B**) After recovery and plating on solid media with rifampicin, the molecular identity of the nearby synonymous mutation (highlighted nearby) appears to have a minimal effect on the efficiency of introducing Rif^R^ phenotype, even if the nearby mispair is NucS-active (*). (**C**) Nearby NucS-active mismatches only cause only a minor growth defect in survival caused by a NucS-inactive mismatch generating rifampicin resistance, not an orders-of-magnitude drop as expected in a HR model. Colony forming units (CFUs) were calculated by dividing the number of colonies appearing on solid media with rifampicin after transfection with the oligonucleotides and recovery by the number of colonies appearing on solid media with kanamycin (which they have by virtue of plasmid pJV62 used to enhance oligonucleotide recombination; see Materials and methods). Error bars are 95% confidence from three replicates. (**D**) From Sanger sequencing of *M. smegmatis* recovered together in liquid media with rifampicin (not isolated as colonies), synonymous mutations caused by NucS-inactive mismatches are correlated with rifampicin-resistance mutation caused by a NucS-inactive mismatch, but synonymous mutations caused my NucS-active mismatches are not and appear to be corrected.

We then performed OR using oligonucleotides that introduced both a (NucS-inactive) dA-dC mismatch to produce rifampicin resistance if unrepaired (*rpoB* c.1327A > G (p.H442R)) and a nearby (NucS-active) dG-dT mismatch that would generate a synonymous mutation in *rpoB* if unrepaired (Figure 3). Based on the model that a DSB caused by NucS at a mismatch would be repaired by HR (16), we expected that growth after transfection with those oligonucleotides would resemble those containing dG-dT mismatches in Figure 2, that is, having orders of magnitude fewer survivors compared to those introducing a dA-dC mismatch alone, because we would expect the nearby dA–dC mismatch to be repaired ‘collaterally’ by gene conversion or end resection at the initiation of HR (23). However, *M. smegmatis* that had oligonucleotides to introduce dG–dT mismatches as close as 5 or 6 bp away from the dA–dC mismatch also showed enhanced levels of rifampicin resistance, similar to oligonucleotides that introduced dA-dC mismatches alone (Figures 4A, B, and S3) with only a moderate reduction in the number of rifampicin resistant survivors (Figures 4C and S4) from 2.7 × 10^7^ cfu/ml (±3.5 × 10^6^ cfu/ml, 95% confidence) to between 6.6 × 10^6^ cfu/ml (±3.5 × 10^6^ cfu/ml, 95% confidence) and 1.1 × 10^7^ cfu/ml (±6.9 × 10^6^ cfu/ml, 95% confidence), or 25–40% of the Rif^R^ population without the NucS-active mismatch, compared to fewer than 2000 cfu/ml for the negative control (with no Rif^R^-generating mismatch) (three replicates). Sanger sequencing of the population (kept in liquid media containing rifampicin without plating) confirmed that the rifampicin resistance was largely a result of the specific *rpoB* c.1327A > G mutation introduced *via* the dA–dC mismatch, while there was no evidence of the other dA-to-dG mutations that would have been introduced by the dG–dT mismatch on the same oligonucleotide (Figure 4D). In contrast, if an oligonucleotide that would introduce two NucS-inactive mismatches (dA–dC for rifampicin resistance and dA–dG for a synonymous mutation in *rpoB*), the levels of mutation at both those sites were correlated.

While successful OR itself occurs with low frequency, the above result meant that we could select for the rifampicin resistance mutation caused by a NucS-inactive dA–dC mismatch to isolate and enrich genetic material with successful oligonucleotide incorporation. This would allow us to quantify the presence or absence of synonymous mutations nearby that would also be introduced on the same oligonucleotide (which we hypothesize will not significantly affect bacterial fitness over the course of the experiment) and increase sequencing depth for next-generation sequencing so that we could perform experiments using multiple oligonucleotides with higher throughput. This experimental framework would then allow us to directly compare the mutational results from different variations of oligonucleotides within the same experiment. By filtering the sequencing results based on the presence of the specific NucS-inactive Rif^R^ mutation that we co-introduce (at *rpoB* c.1327A > G), it also allows us to significantly reduce the ‘noise’ in the sequencing results when performing these types of experiments (Supplementary Figure S5) by only evaluating the genetic material of bacteria in which oligonucleotide recombination was successful, as spontaneous Rif^R^ (survivors without successful oligonucleotide recombination) is possible from a variety of other possible mutations within *rpoB*. This in turn allows us to compare our results with strains with *nucS* gene knocked out (43), since the rate of spontaneous Rif^R^ in those strains is significantly higher than in wild-type strains.

**Figure 5.**
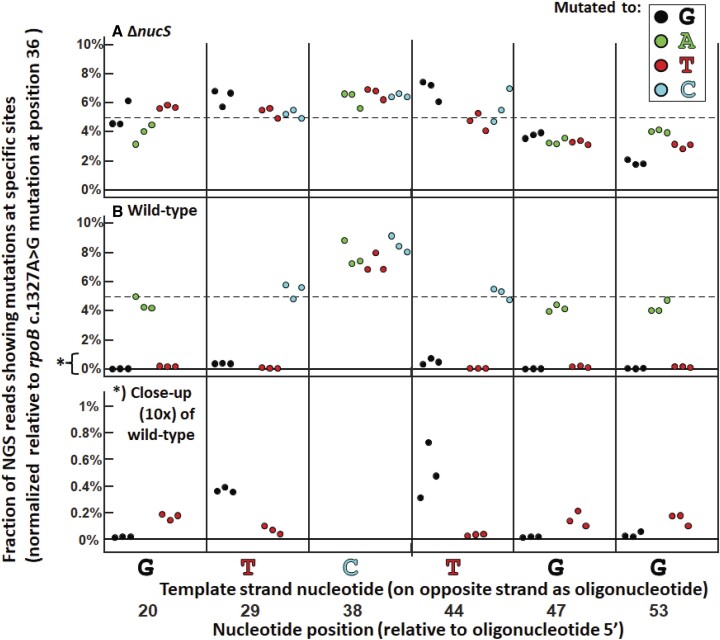
Next-generation sequencing (NGS) results after pooling the oligonucleotides in Figure 3 for OR then transfecting followed by rifampicin selection. The results are from three biological replicates. (**A**) In a *M. smegmatis* strain with nucS gene deleted, each mutation caused by all of the oligonucleotides are represented at approximately equal frequencies (around 5%, as expected for 20 oligonucleotides; dotted line). (**B**) In the wild-type strain, frequency of mutations that would occur as a result of dG–dG, dT–dG and dT–dT mismatches sharply decreases. Below: highlighting low frequency mutations: it appears dT–dT = dG–dT > dT–dG > dG–dT with regards to repair efficiency by NucS.

Repeating this experiment by transfecting a pooled set of the oligonucleotides described in Figure 3, which introduce a rifampicin-selective dA–dC mismatch flanked at various positions by different synonymous mismatches, and compared the results between wild-type and a *nucS*-knockout stain of *M. smegmatis* (Figures 5 and S5) using next-generation sequencing (NGS). The sequencing results confirmed that only the dG–dG, dG–dT and dT–dT mismatches on those oligonucleotides were removed in a NucS-specific manner, while mutations resulting from other mis-pairings remained. The results were highly reproducible and revealed a slight but detectable defect in the repair of dG–dT mismatches relative to dG–dG and dT–dT mismatches, the mutations caused by which were essentially undetected. The results also revealed a slight difference between dT–dG (dT base-paired with template dG) and dG–dT (dG base-paired with template dT), but overall confirmed that the measured biochemical activity of NucS matched the repair of mismatches by *M. smegmatis in vivo* (14,15). Therefore, we conclude that NucS-active mismatches introduced during OR are being specifically removed by a NucS-mediated mechanism.

Surprisingly, there also did not appear to be any effects of changing the site of the NucS-active mismatches to be either 5′- to 3′- relative the dA–dC, as one might expect during repair by HR (i.e. see Figure 7): considering the cleavage pattern of NucS (nicking the DNA two nucleotides 5′- the mismatch on both strands) (14,15,17), one might expect that depending on whether or not the NucS-active mismatch was 5′- or 3′- of the *rpoB* c.1327A > G it might be repaired differently during single crossover HR, on either different fragments or on the same fragment. In these and subsequent experiments, we also found no evidence of the hallmarks of other major double-strand break (DSB) repair mechanism by non-homologous end-joining (NHEJ), that is, no nucleotide insertions or deletions at the site of the repaired mismatches (Supplementary Figure S6) (44).

**Figure 6.**
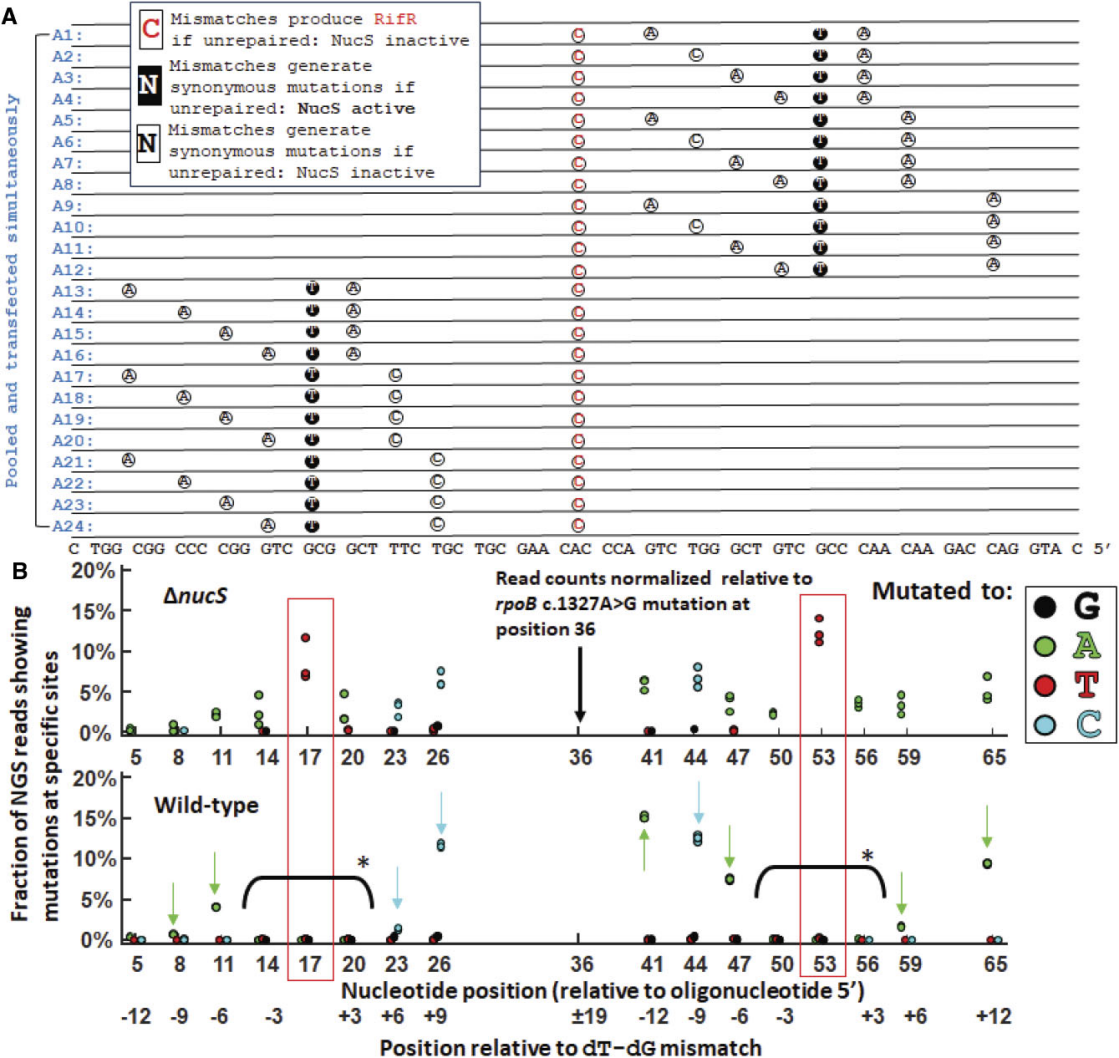
NucS-associated MMR collaterally repairs NucS-inactive mismatches within six nucleotides of a NucS-active mismatch but not outside a 6–9 nt region of the NucS-active mismatch. (**A**) Pooled oligonucleotides (blue, see Figure 2 caption) that contain (i) a dA–dC mismatch that should introduce a rifampicin resistant phenotype if unrepaired; (ii) a NucS-active dT–dG mismatch located either 5′- or 3′- of (i) that would produce synonymous mutation if unrepaired and (iii) two NucS-inactive mismatches (e.g. dA–dC, dC–dC, dA–dA, dT–dC) that would produce synonymous mutations in rpoB if unrepaired, at various positions relative to (i) and (ii). (**B**) Mutations generated by both NucS-active (boxed in red) and NucS-inactive mismatches within 3 nt of a NucS-active mismatch are significantly depleted in the NucS-active strain (marked by asterisk). Mutations generated by NucS-inactive mismatches >6 nt away are largely unaffected (marked by arrows), though there is a slight effect 9 nt 3′- of the NucS-active mis-pair. Note that the results presented show three biological replicates (if fewer than three dots are observed, it is because they are overlapping). Supplementary Figure S7 has a variation of this experiment.

We then performed OR using a pooled set of oligonucleotides that would introduce (Figures 6A and S7):

- a dA–dC mismatch that should introduce a rifampicin resistant phenotype if unrepaired;
- a NucS-active dT–dG mismatch located either 5′- or 3′- of (i) that would produce synonymous mutation if unrepaired; and
- one or more NucS-inactive mismatches (e.g. dA–dC, dC–dC, dA–dA, dT–dC) that would produce synonymous mutations in *rpoB* if unrepaired, at various positions relative to (i) and (ii).

**Figure 7.**
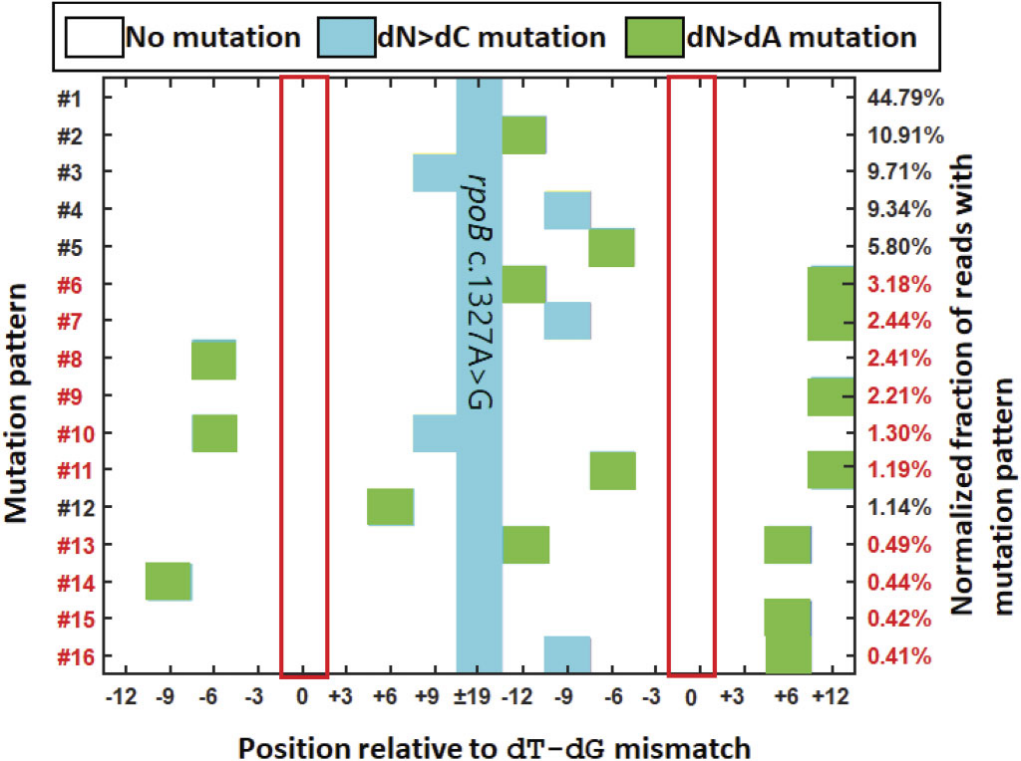
Mutation patterns from next-generation sequencing (NGS) with relative frequency >0.1%, the average of three biological replicates, observed after OR with pooled oligonucleotides from Figure 6A. Red boxes highlight the position of the dT–dG mismatches. Patterns marked with red labels have mutations introduced to both sides the site of the dT–dG mismatch, with no associated dC > dT mutation. Note that for 24 pooled oligonucleotides, the expected number of reads per oligonucleotide is ∼4.2%.

After selection in rifampicin and NGS, we report several unexpected findings. As before, in a NucS-dependent manner we found that the dT–dG mismatches were efficiently repaired when flanked by NucS-inactive nucleotides, but that NucS-inactive mismatches were found to be repaired ‘collaterally’ when located 3 nt away from the mismatched dT–dG (Figures 6B and S6). Mutations caused by a dT–dG mis-pair, along with mutations caused by dA–dG mis-pairs located 3 nt away in either direction from the dT–dG site, were significantly depleted from sequencing reads in wild-type (NucS active) *M. smegmatis*. This was observed in both cases where the dT–dG mismatch is located either 5′- and 3′- of the *rpoB* c.1327A > G site, and in all oligonucleotides. However, if the mis-matched nucleotides flanking the dT–dG mis-pair were located located 6 nt away or farther from the site of the dT–dG mis-pair, they were largely not repaired, although there appeared to be an partial collateral repair of 3′- but not 5′- NucS-inactive mis-pairs located 6 nt away (but not the one 9 nt away). When looking at the correlation between mutations that are observed in the sequencing reads (Figure 7), we find a substantial fraction of all of the NGS (∼15%, patterns #6–11 and #13–16) showed mutations both 5′- and 3′- the site of the repaired dT–dG mis-pair—and indeed located as close as 6 nt away from the site of the dT–dG mis-pair—but no mutations caused by the dT–dG mis-pair. This would imply that a NucS-associated MMR process can occur within a notably short patch of DNA.

The distribution of the occurrences of these mutational patterns (Figure 7) also suggests that NucS-mediated repair might stochastically extend slightly beyond 6 nt, with the probabilities of extending beyond that range sharply decreasing with increasing distance from the NucS-active mis-pair. For example, pattern #1 would be expected to be observed after repair for both oligos A4 and A14 (Figure 6A), which only have mis-pairs located 3 nt away from the dT-dG mis-pair, and patterns #2–#5 and #16 would be expected to be observed with oligos A1, A24, A2, A3 and A20, respectively, each of which having one of their NucS-inactive mis-pairs located within 3 nt away from the dT-dG mis-pair. From 24 pooled oligonucleotides, one would expect on average 4.2% of all of the NGS reads to originate from each oligo so, for example: comparing the prevalence of pattern #2 (10.91%), pattern #6 (3.18%), and pattern #13 (0.49%), which have mutations 12 bp 5′- of the dT-dG mis-pair and either no mutation observed (#2), a mutation 12 bp 3′- of the site of the dT-dG mis-pair (#6), and a mutation 6 bp 3′- of the site of the dT–dG mis-pair (#13), respectively, one can see a decrease in the observation of mutations 3′- of the site of the dT–dG mis-pair with increased proximity to the site of the dT–dG mis-pair, as oligo A5 would generate pattern #2 if the 3′- mis-pair is collaterally repaired instead of pattern #13 and oligo A13 would also generate pattern #2 if the 3′- mis-pair is collaterally repaired instead of pattern #6. A similar trend is also observed when examining the prevalence of patterns #4 (9.34%), pattern #7 (2.4%) and pattern #16 (0.41%), which have mutations 9 bp 5′- of the dT–dG mis-pair and either no mutation observed (#2), a mutation 12 bp 3′- of the dT–dG mutation (#7), and a mutation 6 bp 3′- of the dT–dG mutation (#16), respectively. Likewise for pattern #5 (5.8%) and pattern #11 (1.19%), with a mutation 6 bp 5′- of the dT–dG mis-pair and either no observed (#5) or one observed mutation 12 bp 3′- of the dT–dG mis-pair (#11). A stochastically-varying repair patch of short but varying size, largely restricted to within 6 nt but sometimes extending beyond in either direction, might explain the marginal but statistically-significant defect in Rif^R^ colonies observed when synonymous NucS-active and Rif^R^-generating NucS-inactive mis-pairs are co-introduced (Figure 4C) if the Rif^R^-generating NucS-inactive mis-pair are also collaterally repaired with some probability along with the nearby NucS-active mis-pair (either 6 bp or 15 bp between). However, because there is only a moderate drop in the rate of Rif^R^-generation rather than an orders-of-magnitude decrease, it would suggest that the repair patches far larger than 6 bp are relatively rare. Understanding the mechanism that regulates the size of the short repair patch during NucS-mediated repair remains an open question.

**Figure 8.**
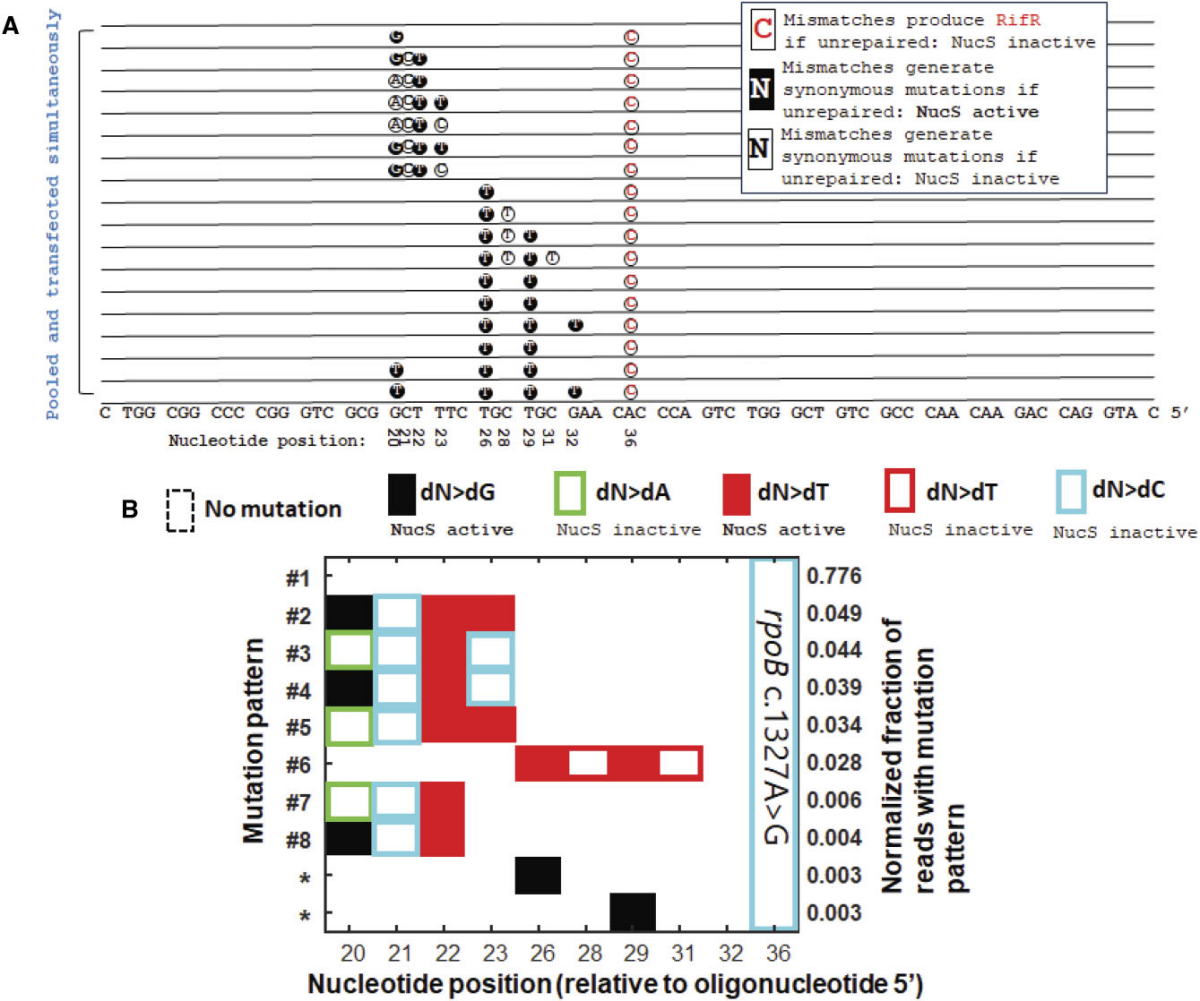
Clustered mismatches are best able to allow NucS-active mismatches to evade repair during OR gene editing of *M. smegmatis*. (**A**) Sequences of the pooled oligonucleotides evaluated for mutational patterns after rifampicin resistance. (**B**) Mutational patterns (with relative frequency > 0.1%) observed after sequencing. Oligonucleotides that introduce four consecutive mismatches, whether or not they are NucS-active or inactive, appear at the highest frequency, followed by four mismatches in across six nucleotides, followed by three consecutive mismatches. Patterns labelled with ‘*’ represent unexpected mutational patterns observed that were not intentionally introduced by OR.

Lastly, in other species, OR can be performed in a way to evade DNA MMR and increase OR efficiency for mis-pairs that would otherwise be very readily repaired before mutations were made permanent (25,37). This is done by exploiting the inability of MutS and MutS homologues to recognize dC–dC mismatches or complex lesions, and it had been previously found that introducing complex mismatches, where multiple dC–dC mismatches flank the mismatch of interest at locations where they would generate synonymous mutations, or a series of multiple consecutive mis-pairs, could be used to increase OR efficiency of mutations caused by MutS-recognized mismatches. Because *M. smegmatis* NucS has a different spectrum of mismatches recognized than MutS, we investigated which types of complex lesions might evade NucS-mediated repair by introducing a pooled set of oligonucleotides containing a various combinations of lesions clustered together on the same oligonucleotide (Figure 8A). We found that introducing three consecutive or semi-consecutive lesions within 6 nt of each other near a NucS-active mismatch, whether or not they are NucS-active or inactive, appeared to be able to most efficiently evade NucS-mediated repair even when including, dG–dG, dG–dT, dT–dT mismatches (Figure 8B).

## Discussion

**Figure 9.**
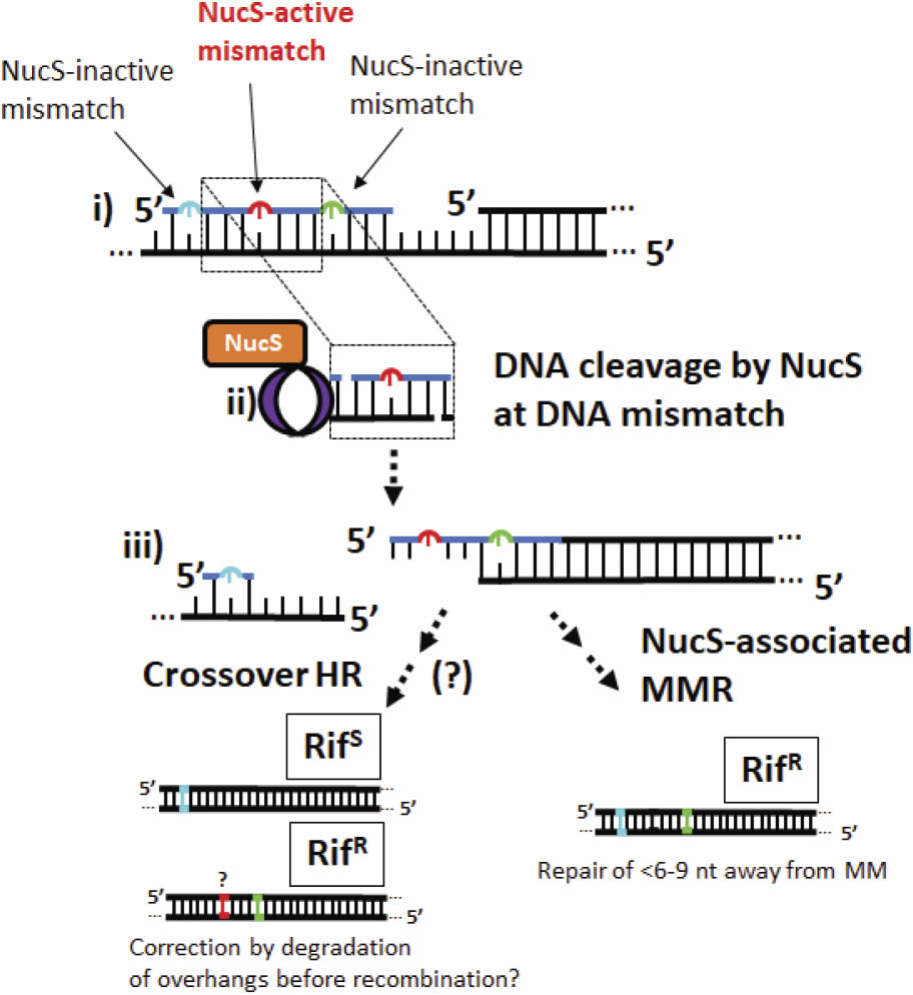
Summary of experimental results. (i) If during OR an oligonucleotide introduces a NucS-active dT–dG mismatch (red) to introduce a synonymous mutation is flanked at short distance by a NucS-inactive mutation to produce a synonymous mutation (blue) and NucS-inactive mutation that causes resistance to rifampicin (yellow), (ii) NucS (orange) with beta clamp (purple) cleaves the dT–dG mismatch with two strand breaks located 2 nt 5′- of the mismatched dT and dG. (iii) If repaired by a homologous recombination (HR)-like mechanism, we would not expect both of the mutations caused by the flanking mismatches to appear together with single-crossover repair events. However, in a substantial number of cases we find both flanking mutations with no corresponding mutation caused by the aggravating dT in between them.

Our work, where genomic mismatches are introduced specifically into the genome of *M. smegmatis*, provides evidence that NucS is involved in the direct repair of dT-dG mismatches *in vivo*, rather than, as proposed, promoting the death of cells that are hyper-mutative for other reasons. Flanking NucS-inactive mismatches on the same oligonucleotide as a NucS-stimulating mismatch remain unrepaired and are largely converted to permanent genetic mutations if they are located >5 bp away from a NucS-stimulating mismatch, which suggests that the repair of the NucS-stimulating mismatch is confined within that small region. *In vitro*, NucS cleaves both strands two nucleotides 5′- of the mismatch (14,15,17), generating short sticky ends (Figures 9i and ii), but we also observe NucS-inactive mismatches repaired ‘collaterally’ if located three nucleotides away from NucS-activating mismatches and, with some probability, slightly beyond that.

These findings are difficult to reconcile with the model of canonical RecA-dependent mycobacterial HR: they would not be observed for single-crossover HR (Figure 9iii), and a double-crossover tract would be required to observe such a mutational pattern—and that tract would have to be extraordinarily short; it would be short even in terms of fundamental limits of RecA filament assembly and homology recognition (45). The outcomes of repair more closely resemble repair by the mycobacterial NHEJ pathway (23,44,46), but with several differences: (i) NHEJ tends to be highly mutagenic, including with insertions and deleted nucleotides at the site of repair, which we do not observe. Despite multiple possibilities for synonymous mutations at the sites of the dT–dG mismatches, we do not observe elevated mutation rate at that site to other nucleotides, but instead only correct repair to dC. (ii) There is no known mechanism for ‘strand discrimination’ in the canonical NHEJ: that is, in the absence of discriminating between 5′- overhangs caused by NucS, if one or the other 5′- overhang was being used to template repair, we might expect 50% repair of dT and 50% incorrect ‘repair’ of the dG at dT–dG mispairs. In fact, we observe a near-total absence of dT mutations at the sites of dT–dG mismatches, the dT having been corrected back to dC. These findings would all suggest that NucS is involved in a mechanistically-distinct MMR/DSB repair reaction that occurs within a short patch (Figure 9). Recently, Rivera-Flores *et al.* reported that knock-out of the genes for homologues of recombinases RecA and RadA (involved in mycobacterial homologous recombination), for homologues of Ku, LigD and FenA (involved in mycobacterial non-homologous end-joining), and for combinations thereof did not affect mismatch repair by NucS (47), further supporting our conclusions that NucS-mediated MMR is differentiated from other mycobacterial DSB repair pathways.

We can note that our findings do not strictly eliminate the possibility of a NucS-mediated repair of mismatches that is mediated by a HR-like mechanism or other DSB mechanisms that might also be present, and it is possible that degradation of the overhangs caused after NucS cleavage could result in complete repair using a sister chromatid with some unidentified recombinase (47). Mycobacterial helicase-nuclease AdnAB (48) that participates in HR has been shown to ‘nibble’ a few 5′- nucleotides from sticky ends (49), and perhaps this is performed prior to a HR-like mechanism after cleavage by NucS to remove both new and template sequences specifically at the site of mis-pairings. Repair by a HR-like mechanism (Figure 9iii) that collaterally eliminates the Rif^R^-generating mis-pair through gene conversion, and/or the stochastic extension of the short patch of repair during NucS-mediated MMR that we find evidence of in the mutational patterns from NGS reported in Figure 7, may account for the moderate decrease in Rif^R^ mutants we observe when oligonucleotides that introduce both NucS-stimulating mis-pairs are included along with sites that introduce NucS-inactive Rif^R^-generating mis-pairs. Determining what regulates the size of the short-patch and what other proteins are involved will be key to understanding the mechanism of NucS-mediated MMR.

A potential limitation of this study is that we were selecting for mutations in *rpoB*, an essential mycobacterial gene, and as such the repertoire of mutations that we would have the ability to observe by sequencing after repair—for example, frameshifts or large deletions—are less like to be observed if we were performing this assay in a nonessential gene (23). That is, while genetic mutation with the *rpoB* is an important cause of antibiotic resistance in *M. tuberculosis* and other mycobacterial pathogens (11), performing OR within *rpoB* could potentially constrain the gene editing outcomes we can observe in this study, as those bacteria would not survive to be sequenced. Another potential explanation for the (relatively minor) reduction (to ∼25% to 40%) of Rif^R^ mutants observed when oligonucleotides are used that introduce NucS-active mis-pairs along with a NucS-inactive mis-pair that generates Rif^R^ (Figure 4B) is that this reduction is a result of bacterial cell death occurring when these frameshifts or large deletions in *rpoB* do occur during repair. However, when comparing that modest reduction to the near-100% rate of repair of NucS-active mis-pairs that we observe in the survivors, this would suggest that a short-patch NucS-mediated MMR is at least as important means of repairing mismatches *in vivo* as ones that might introduce larger mutations, frameshifts, or repair of longer patches of DNA that might also collaterally prevent the mutations for Rif^R^ from remaining permanent.

Having established OR as an experimental tool that can be used to probe mechanisms of NucS-mediated mutation avoidance in mycobacteria, and demonstrated ways to improve OR for mycobacterial genome engineering, it will be interesting to combine with other genetic tools (26,50–52) to further deconstruct the mechanism of mycobacterial MMR.

## Supplementary Material

gkae402_Supplemental_File

## Data Availability

Sequencing data underlying this article is available at NCBI Bioproject (https://www.ncbi.nlm.nih.gov/bioproject/) under accession code PRJNA1105094. Scripts for analysis are available at https://doi.org/10.5281/zenodo.11085873.
