# Supplementary material for: Genome editing outcomes reveal mycobacterial NucS participates in a short-patch repair of DNA mismatches
Source: Nucleic Acids Res. 2024 May 15;52(20):12295–307. doi: 10.1093/nar/gkae402 (PMC11551744; doi:10.1093/nar/gkae402)
Supplement: gkae402_Supplemental_File [file gkae402_supplemental_file.pdf]

Genome Editing Outcomes Reveal Mycobacterial NucS Participates in a Short-Patch Repair of DNA Mismatches

AUTHORS

Tanjina Islam[1] and Eric A. Josephs[1,2,*]

[1] Department of Nanoscience, University of North Carolina at Greensboro, Greensboro, NC, 27401, USA

[2] Department of Biology, University of North Carolina at Greensboro, Greensboro, NC, 27401, USA

* To whom correspondence should be addressed. Tel: +1 336 285 2890; Email: eric.josephs@uncg.edu

SUPPORTING INFORMATION

Figure S1. All three replicates of the experiment described in Figure 2.

Figure S2. Estimates of the frequencies of rifampicin-resistance after electroporation.

Figure S3. Additional replicates of experiment described in Figure 2B (top) and 2C (bottom), respectively.

Figure S4. Serial dilutions of *M. smegmatis* plated on rifampcin after oligonucleotide recombination (3 replicates) using oligos described in Figure 3.

Figure S5. Fraction of reads with mutations in rpoB, after filtering for the presence of the selectable Rif^R mutation that we co-introduce at *rpoB* c.1327A>G, related to Figure 5.

Figure S6. Histogram of read lengths between the sequences immediately outside the oligonucleotide sequences.

Figure S7. Variation of experiment performed in Figure 6, again showing that NucS-associated MMR collaterally repairs NucS-inactive mismatches within 6 nucleotides of a NucS-active mismatch but not outside 6 – 9 nt of the NucS-active mismatch.

Table S1: List of oligonucleotides

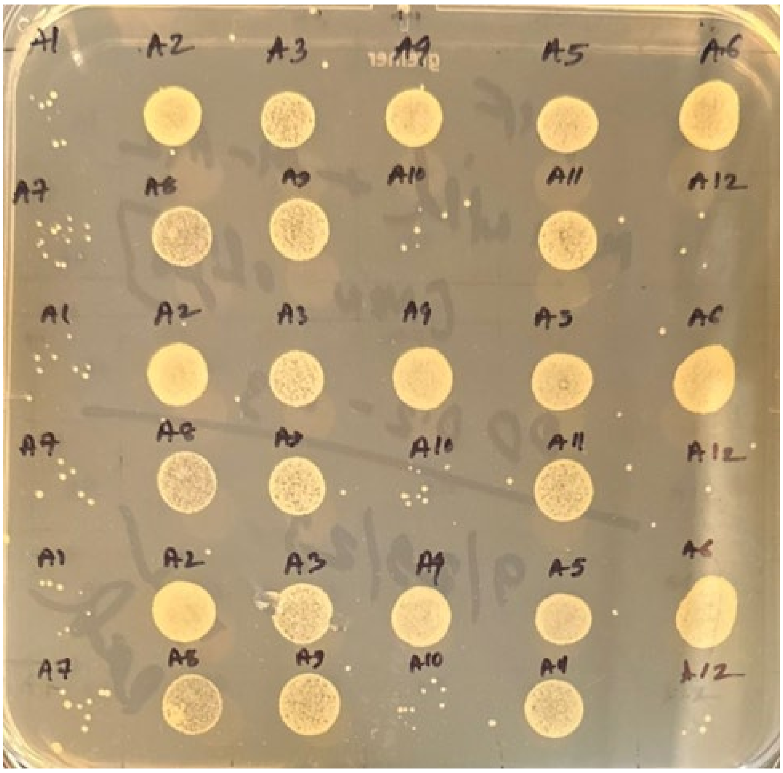

Figure S1. All three replicates of experiment described in Figure 2.

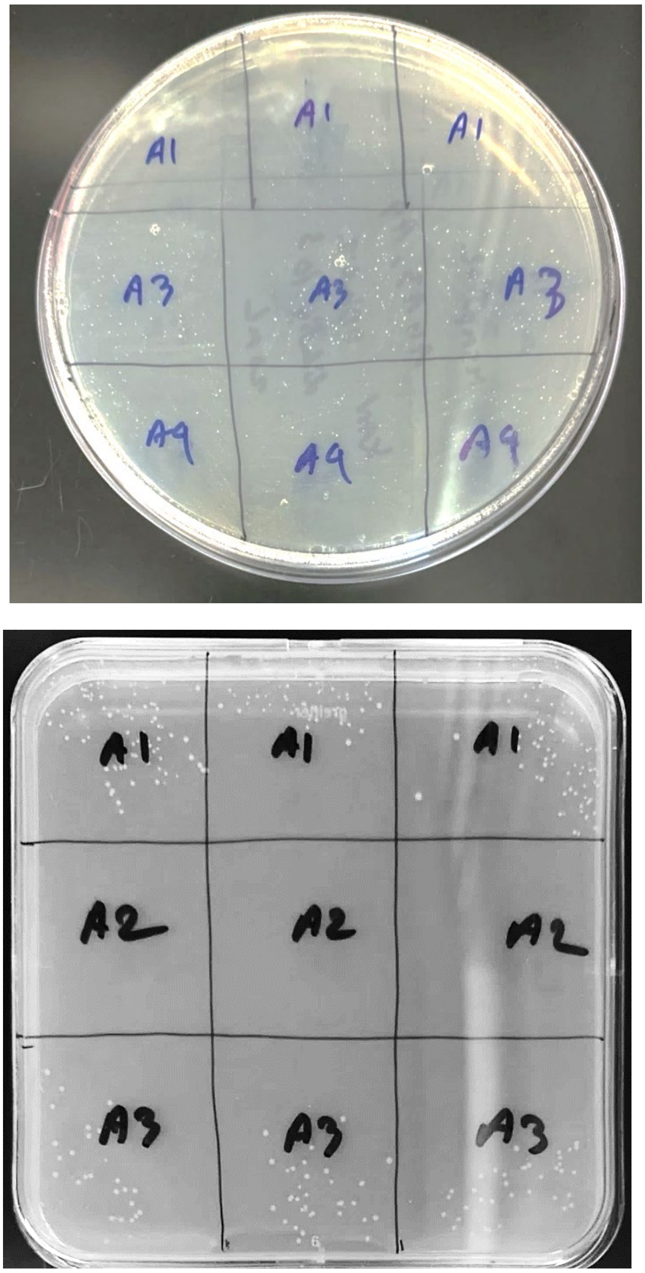

Figure S2. Estimates of the frequencies of rifampicin-resistance after electroporation. After electroporation with oligonucleotides (NOTE: oligos are labelled as described in Figure 3 and S3, with oligo A1 introducing a single RifR mutation and oligo A2 introducing no mutations as a negative control, and oligo A3 introducing two mutations including the RifR mutation), *M. smegmatis* were diluted 1000x and 15 uL plated on plates containing (top) kanamycin (50 ug/mL), with resistance by virtue of plasmid pJV62, and (bottom) rifampicin (25 ug/mL), with resistance from of mutations introduced into the *rpoB* gene. By colony count, RifR efficiency appears to be 28% of KanR for oligo A3.

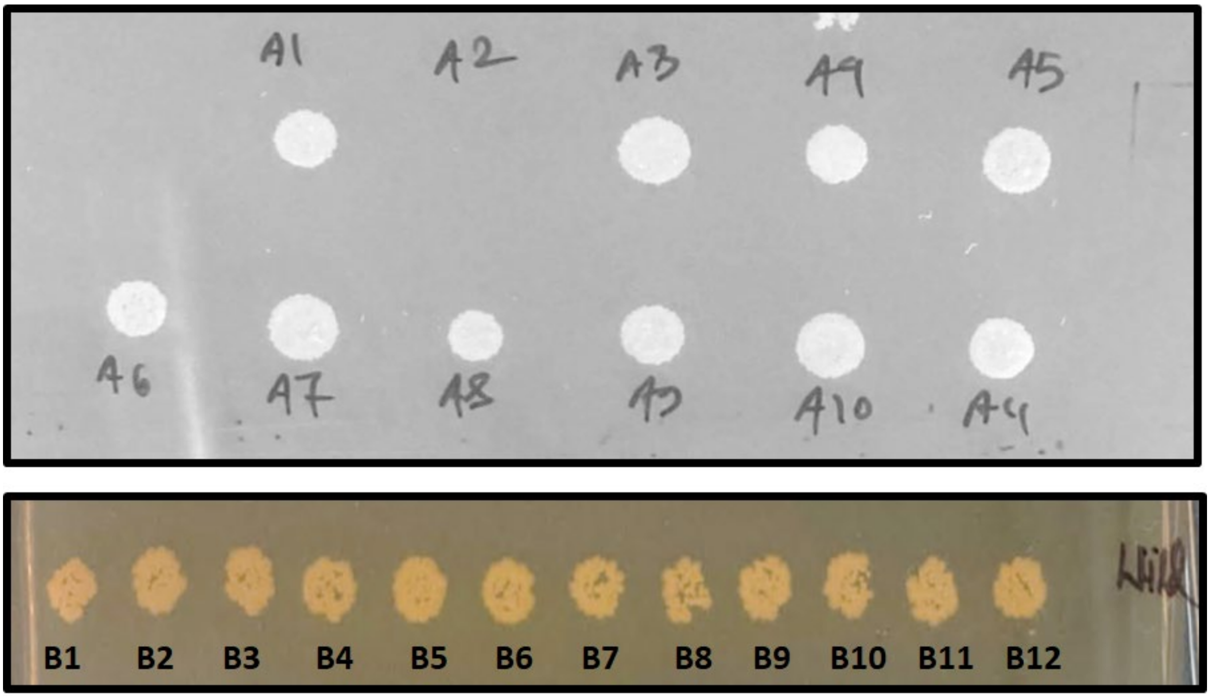

Figure S3. Additional replicates of experiment described in Figure 2B (top) and 2C (bottom), respectively.

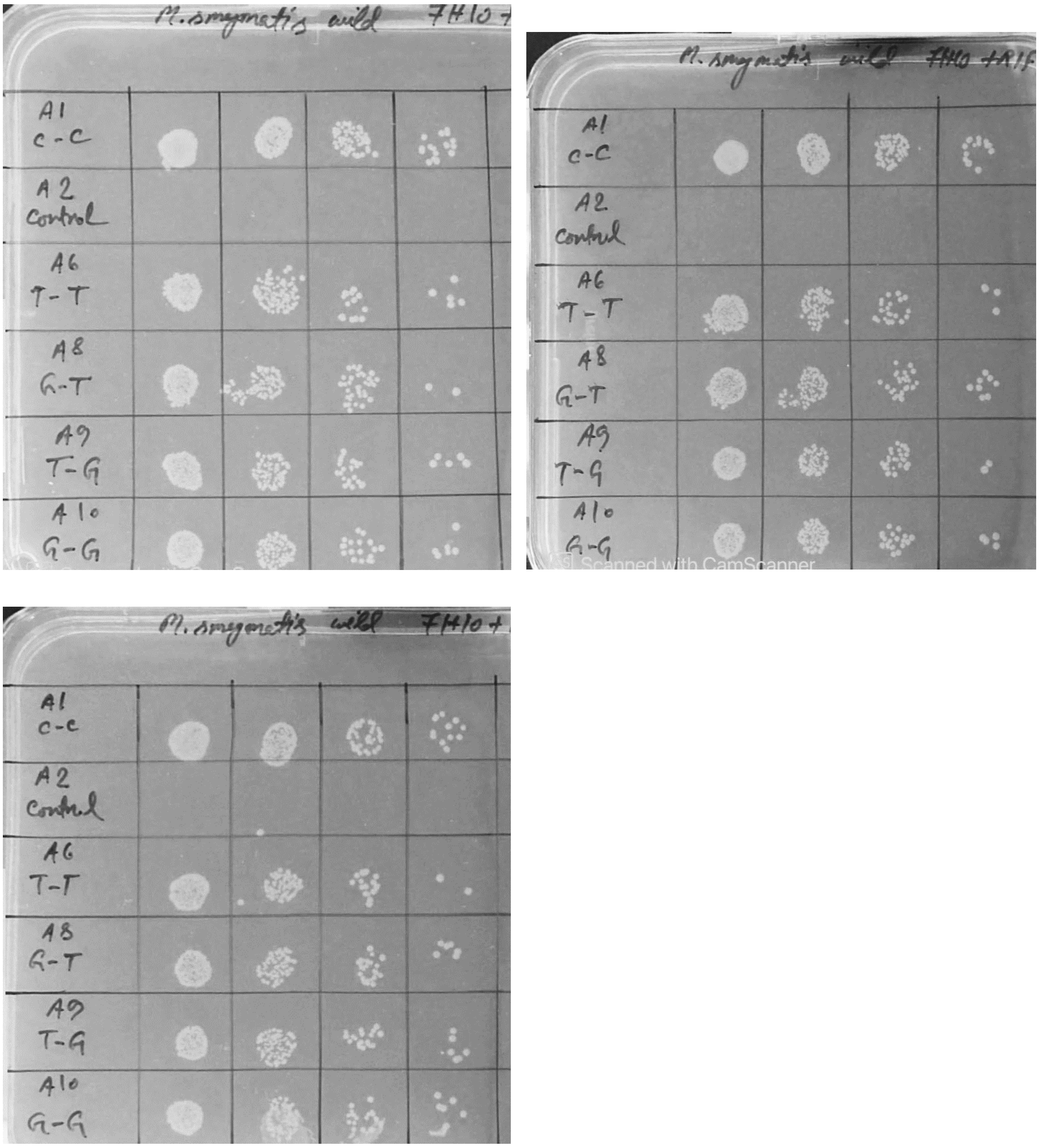

Figure S4. Serial dilutions of *M. smegmatis* plated on rifampcin after oligonucleotide recombination (3 replicates) using oligos described in Figure 3. 5 ul of recovered culture (described in methods) were plated after being diluted (left to right) 10x, 100x, 1000x, and 10000x.

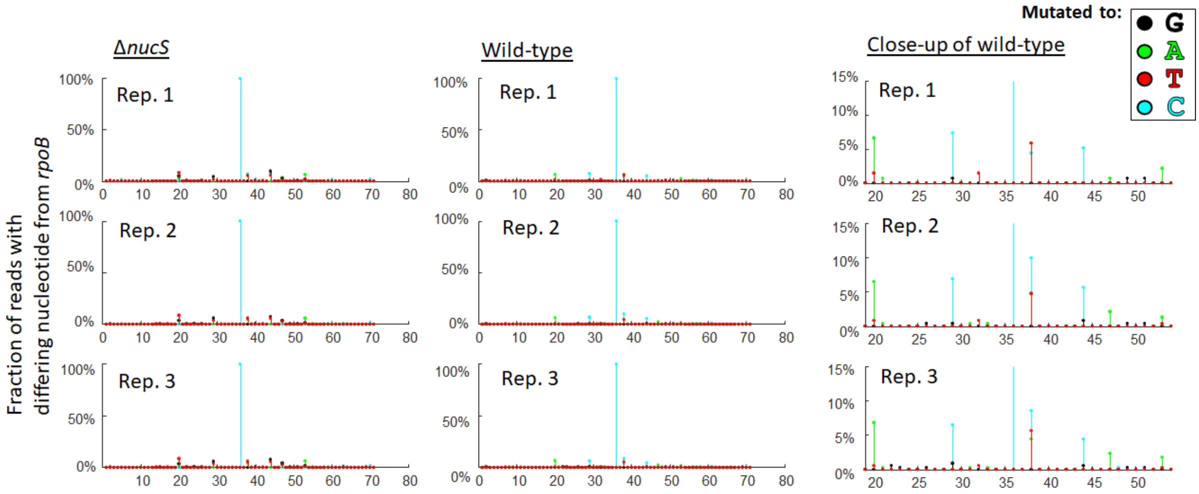

Figure S5 Fraction of reads with mutations in rpoB, after filtering for the presence of the selectable Rif^R mutation that we co-introduce at *rpoB* c.1327A>G. This is the 'full' version of Figure 5. After identifying the presence of the sequences flanking the oligonucleotide targeted sequence, if the length of sequences between those regions was the expected 71 bp and the forward and reverse reads were identical, it was determined whether or not there were any nucleotides that differed from the *rpoB* sequence. Those sequences were only further analysed if the *rpoB* c.1327A>G (here shown as a mutation to dC as the oligonucleotides introduces a dC-dA mismatch). As can be seen, there is very little noise (mutations outside of the specific sites where they are introduced during oligonucleotide recombination), even for the *nucS*-knockout strain.

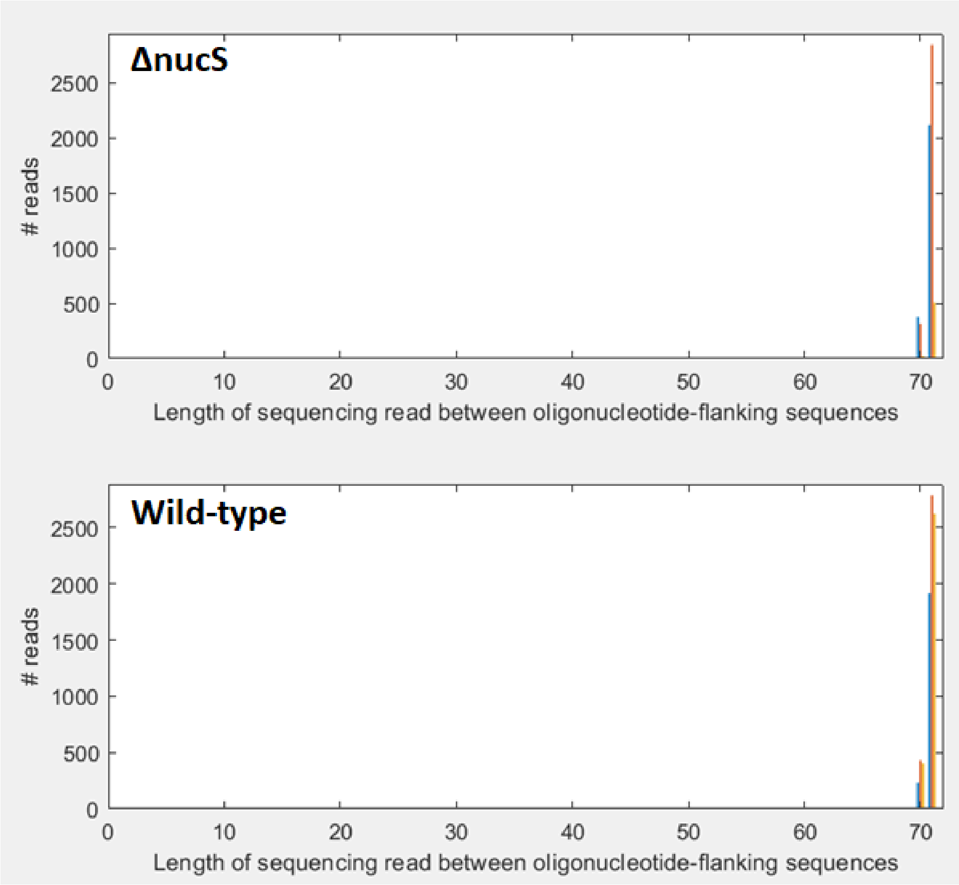

Figure S6 Histogram of read lengths between the sequences immediately outside the oligonucleotide sequences. Expected length 71 bp. Note there is a small amount of sequences that appear to be 70 bp: these appear in both NucS knockout and wild-type strains, and are very likely a sequencing artifact of a 'missing' nucleotide in a region of low complexity, away from the sites of introduced mutations. There is no evidence of insertions or deletions during repair. Different colors are the 3 replicates.

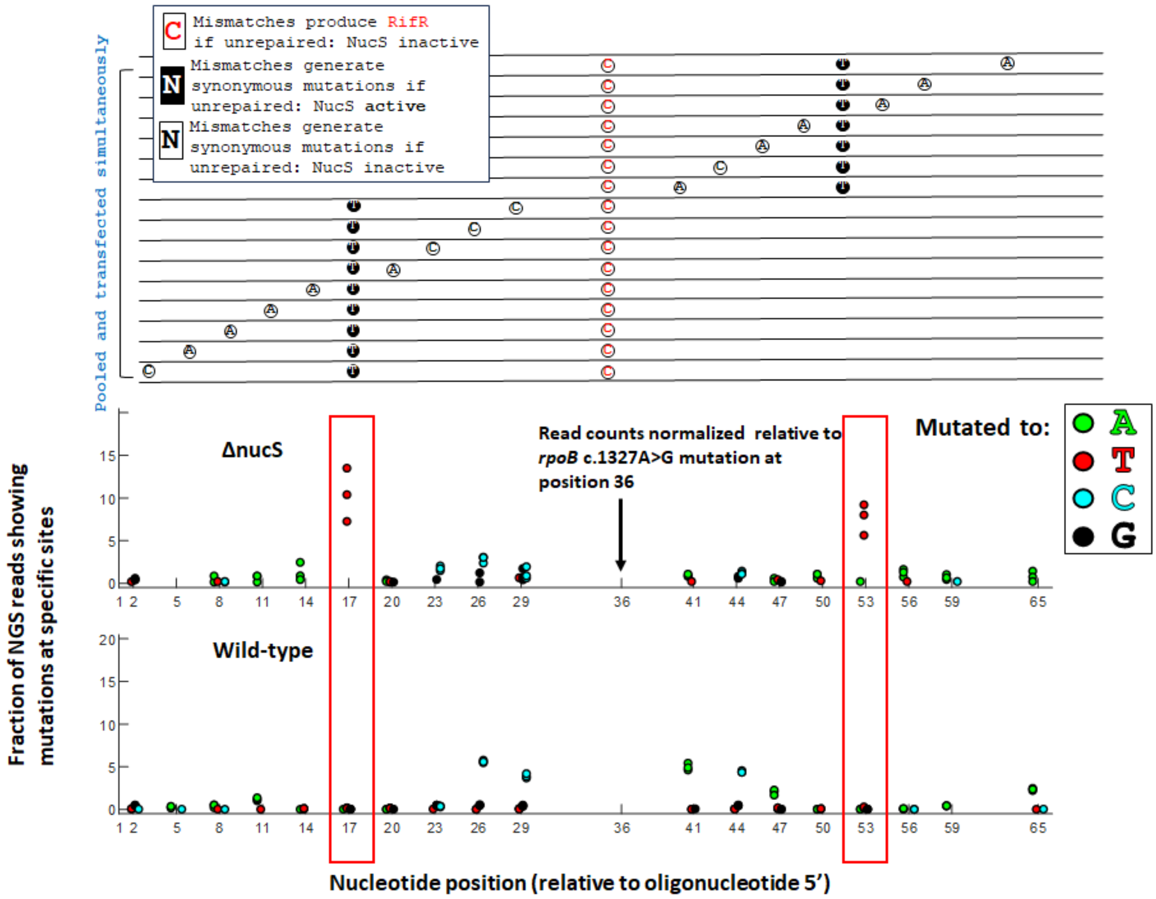

Figure S7. Variation of experiment performed in Figure 4, again showing that NucS-associated MMR collaterally repairs NucS-inactive mismatches within 6 nucleotides of a NucS-active mismatch but not outside 6 – 9 nt of the NucS-active mismatch**.** A) Pooled oligonucleotides (blue, see Figure 1 caption) that contain i) a dA-dC mismatch that should introduce a rifampicin resistant phenotype if unrepaired; ii) a NucS-active dT-dG mismatch located either 5′- or 3′- of (i) that would produce synonymous mutation if unrepaired; and iii) one NucS-inactive mismatches (e.g., dA-dC, dC-dC, dA-dA, dT-dC) that would produce synonymous mutations in rpoB if unrepaired, at various positions relative to (i) and (ii). B) Mutations generated by both NucS-active (boxed in red) and NucS-inactive mismatches within 3 nt of a NucS-active mismatch are significantly depleted in the NucS-active strain. Mutations generated by NucS-inactive mismatches > 6 nt away are largely unaffected, though there is a slight effect 9 nt 3′- of the NucS-active mis-pair. Note that the results presented show 3 biological replicates (if fewer than three dots are observed, it is because they are overlapping).

Table S1: List of oligonucleotides

**Related to Figure 1**	
WT	GACCGCCGGGGCCCAGCGCCGAAAGACGACGCTTGTGGGTCAGA CCCGACAGCGGGTTGTTCTGGTCCATG
H442D,C-C	GACCGCCGGGGCCCAGCGCCGAAAGACGACGCTTGTCGGTCAGA CCCGACAGCGGGTTGTTCTGGTCCATG
H442L,A-A	GACCGCCGGGGCCCAGCGCCGAAAGACGACGCTTGAGGGTCAGA CCCGACAGCGGGTTGTTCTGGTCCATG
H442P,G-A	GACCGCCGGGGCCCAGCGCCGAAAGACGACGCTTGGGGGTCAGA CCCGACAGCGGGTTGTTCTGGTCCATG
H442R,C-A	GACCGCCGGGGCCCAGCGCCGAAAGACGACGCTTGCGGGTCAGA CCCGACAGCGGGTTGTTCTGGTCCATG
H442Y,A-C	GACCGCCGGGGCCCAGCGCCGAAAGACGACGCTTGTAGGTCAGA CCCGACAGCGGGTTGTTCTGGTCCATG
R445P,G-G	GACCGCCGGGGCCCAGCGCCGAAAGAGGACGCTTGTGGGTCAGA CCCGACAGCGGGTTGTTCTGGTCCATG
R445L,G-A	GACCGCCGGGGCCCAGCGCCGAAAGAAGACGCTTGTGGGTCAGA CCCGACAGCGGGTTGTTCTGGTCCATG
S447L,C-A	GACCGCCGGGGCCCAGCGCCAAAAGACGACGCTTGTGGGTCAGA CCCGACAGCGGGTTGTTCTGGTCCATG
S447P,G-T	GACCGCCGGGGCCCAGCGCCGGAAGACGACGCTTGTGGGTCAGA CCCGACAGCGGGTTGTTCTGGTCCATG
S447L,C-C	GACCGCCGGGGCCCAGCGCCCAAAGACGACGCTTGTGGGTCAGA CCCGACAGCGGGTTGTTCTGGTCCATG
L449P,G-T	GACCGCCGGGGCCCGGCGCCGAAAGACGACGCTTGTGGGTCAGA CCCGACAGCGGGTTGTTCTGGTCCATG
**Related to Figures 2 and 3**	
JCV254/Pos. control	GACCGCCGGGGCCCAGCGCCGAAAGACGACGCTTGTGGGTCAGA CCCGACAGCGGGTTGTTCTGGTCCATG
T441^C>A	GACCGCCGGGGCCCAGCGCCGAAAGACGACGCTTGCGTGTCAGA CCCGACAGCGGGTTGTTCTGGTCCATG
T441^C>G	GACCGCCGGGGCCCAGCGCCGAAAGACGACGCTTGCGCGTCAGA CCCGACAGCGGGTTGTTCTGGTCCATG
T441^C>T	GACCGCCGGGGCCCAGCGCCGAAAGACGACGCTTGCGAGTCAGA CCCGACAGCGGGTTGTTCTGGTCCATG
R444^T>A	GACCGCCGGGGCCCAGCGCCGAAAGACGTCGCTTGCGGGTCAGA CCCGACAGCGGGTTGTTCTGGTCCATG
R444^T>G	GACCGCCGGGGCCCAGCGCCGAAAGACGCCGCTTGCGGGTCAGA CCCGACAGCGGGTTGTTCTGGTCCATG
R444^T>C	GACCGCCGGGGCCCAGCGCCGAAAGACGGCGCTTGCGGGTCAGA CCCGACAGCGGGTTGTTCTGGTCCATG
S447^G>A	GACCGCCGGGGCCCAGCGCTGAAAGACGACGCTTGCGGGTCAGA CCCGACAGCGGGTTGTTCTGGTCCATG
S447^G>C	GACCGCCGGGGCCCAGCGCGGAAAGACGACGCTTGCGGGTCAGA CCCGACAGCGGGTTGTTCTGGTCCATG
S447^G>T	GACCGCCGGGGCCCAGCGCAGAAAGACGACGCTTGCGGGTCAGA CCCGACAGCGGGTTGTTCTGGTCCATG
G438^T>A	GACCGCCGGGGCCCAGCGCCGAAAGACGACGCTTGCGGGTCAGT CCCGACAGCGGGTTGTTCTGGTCCATG
G438^T>G	GACCGCCGGGGCCCAGCGCCGAAAGACGACGCTTGCGGGTCAGC CCCGACAGCGGGTTGTTCTGGTCCATG
G438^T>C	GACCGCCGGGGCCCAGCGCCGAAAGACGACGCTTGCGGGTCAGG CCCGACAGCGGGTTGTTCTGGTCCATG
S437^G>A	GACCGCCGGGGCCCAGCGCCGAAAGACGACGCTTGCGGGTCAGA CCTGACAGCGGGTTGTTCTGGTCCATG

S437^G>C	GACCGCCGGGGCCCAGCGCCGAAAGACGACGCTTGCGGGTCAGACCGGACAGCGGGTTGTTCTGGTCCATG
S437^G>T	GACCGCCGGGGCCCAGCGCCGAAAGACGACGCTTGCGGGTCAGACCAGACAGCGGGTTGTTCTGGTCCATG
P435^G>A	GACCGCCGGGGCCCAGCGCCGAAAGACGACGCTTGCGGGTCAGACCCGACAGTGGGTTGTTCTGGTCCATG
P435^G>C	GACCGCCGGGGCCCAGCGCCGAAAGACGACGCTTGCGGGTCAGACCCGACAGGGGGTTGTTCTGGTCCATG
P435^G>T	GACCGCCGGGGCCCAGCGCCGAAAGACGACGCTTGCGGGTCAGACCCGACAGAGGGTTGTTCTGGTCCATG
**Related to Figure S4 (coordinates from oligonucleotide 5')**	
(T36>C)(C53>T)	GACCGCCGGGGCCCAGCGCCGAAAGACGACGCTTGCGGGTCAGACCCGACAGTGGGTTGTTCTGGTCCATG
(T36>C)(C53>T)(G65>A)	GACCGCCGGGGCCCAGCGCCGAAAGACGACGCTTGCGGGTCAGACCCGACAGTGGGTTGTTCTGATCCATG
(T36>C)(C53>T)(G59>A)	GACCGCCGGGGCCCAGCGCCGAAAGACGACGCTTGCGGGTCAGACCCGACAGTGGGTTATTCTGGTCCATG
(T36>C)(C53>T)(G56>A)	GACCGCCGGGGCCCAGCGCCGAAAGACGACGCTTGCGGGTCAGACCCGACAGTGGATTGTTCTGGTCCATG
(T36>C)(C50>A)(C53>T)	GACCGCCGGGGCCCAGCGCCGAAAGACGACGCTTGCGGGTCAGACCCGAAAGTGGGTTGTTCTGGTCCATG
(T36>C)(C47>A)(C53>T)	GACCGCCGGGGCCCAGCGCCGAAAGACGACGCTTGCGGGTCAGACCAGACAGTGGGTTGTTCTGGTCCATG
(T36>C)(A44>C)(C53>T)	GACCGCCGGGGCCCAGCGCCGAAAGACGACGCTTGCGGGTCAGCCCCGACAGTGGGTTGTTCTGGTCCATG
(T36>C)(C41>A)(C53>T)	GACCGCCGGGGCCCAGCGCCGAAAGACGACGCTTGCGGGTAAGACCCGACAGTGGGTTGTTCTGGTCCATG
(C17>T)(A29>C)(T36>C)	GACCGCCGGGGCCCAGTGCCGAAAGACGCCGCTTGCGGGTCAGACCCGACAGCGGGTTGTTCTGGTCCATG
(C17>T)(A26>C)(T36>C)	GACCGCCGGGGCCCAGTGCCGAAAGCCGACGCTTGCGGGTCAGACCCGACAGCGGGTTGTTCTGGTCCATG
(C17>T)(A23>C)(T36>C)	GACCGCCGGGGCCCAGTGCCGACAGACGACGCTTGCGGGTCAGACCCGACAGCGGGTTGTTCTGGTCCATG
(C17>T)(C20>A)(T36>C)	GACCGCCGGGGCCCAGTGCAGAAAGACGACGCTTGCGGGTCAGACCCGACAGCGGGTTGTTCTGGTCCATG
(C14>A)(C17>T)(T36>C)	GACCGCCGGGGCCAAGTGCCGAAAGACGACGCTTGCGGGTCAGACCCGACAGCGGGTTGTTCTGGTCCATG
(G11>A)(C17>T)(T36>C)	GACCGCCGGGACCCAGTGCCGAAAGACGACGCTTGCGGGTCAGACCCGACAGCGGGTTGTTCTGGTCCATG
(G8>A)(C17>T)(T36>C)	GACCGCCAGGGCCCAGTGCCGAAAGACGACGCTTGCGGGTCAGACCCGACAGCGGGTTGTTCTGGTCCATG
(G5>A)(C17>T)(T36>C)	GACCACCGGGGCCCAGTGCCGAAAGACGACGCTTGCGGGTCAGACCCGACAGCGGGTTGTTCTGGTCCATG
(A2>C)(C17>T)(T36>C)	GCCCGCCGGGGCCCAGTGCCGAAAGACGACGCTTGCGGGTCAGACCCGACAGCGGGTTGTTCTGGTCCATG
**Related to Figures 4 and 5 (coordinates from oligonucleotide 5')**	
(T36>C)(C50>A)(C53>T)(G65>A)	GACCGCCGGGGCCCAGCGCCGAAAGACGACGCTTGCGGGTCAGACCCGAAAGTGGGTTGTTCTGATCCATG
(T36>C)(C47>A)(C53>T)(G65>A)	GACCGCCGGGGCCCAGCGCCGAAAGACGACGCTTGCGGGTCAGACCAGACAGTGGGTTGTTCTGATCCATG
(T36>C)(A44>C)(C53>T)(G65>A)	GACCGCCGGGGCCCAGCGCCGAAAGACGACGCTTGCGGGTCAGCCCCGACAGTGGGTTGTTCTGATCCATG

(T36>C)(C41>A)(C53>T)(G65>A)	GACCGCCGGGGCCCAGCGCCGAAAGACGACGCTTGCGGGTAAGACCCGACAGTGGGTTGTTCTGATCCATG
(T36>C)(C50>A)(C53>T)(G59>A)	GACCGCCGGGGCCCAGCGCCGAAAGACGACGCTTGCGGGTCAGACCCGAAAGTGGGTTATTCTGGTCCATG
(T36>C)(C47>A)(C53>T)(G59>A)	GACCGCCGGGGCCCAGCGCCGAAAGACGACGCTTGCGGGTCAGACCAGACAGTGGGTTATTCTGGTCCATG
(T36>C)(A44>C)(C53>T)(G59>A)	GACCGCCGGGGCCCAGCGCCGAAAGACGACGCTTGCGGGTCAGCCCCGACAGTGGGTTATTCTGGTCCATG
(T36>C)(C41>A)(C53>T)(G59>A)	GACCGCCGGGGCCCAGCGCCGAAAGACGACGCTTGCGGGTAAGACCCGACAGTGGGTTATTCTGGTCCATG
(T36>C)(C50>A)(C53>T)(G56>A)	GACCGCCGGGGCCCAGCGCCGAAAGACGACGCTTGCGGGTCAGACCCGAAAGTGGATTGTTCTGGTCCATG
(T36>C)(C47>A)(C53>T)(G56>A)	GACCGCCGGGGCCCAGCGCCGAAAGACGACGCTTGCGGGTCAGACCAGACAGTGGATTGTTCTGGTCCATG
(T36>C)(A44>C)(C53>T)(G56>A)	GACCGCCGGGGCCCAGCGCCGAAAGACGACGCTTGCGGGTCAGCCCCGACAGTGGATTGTTCTGGTCCATG
(T36>C)(C41>A)(C53>T)(G56>A)	GACCGCCGGGGCCCAGCGCCGAAAGACGACGCTTGCGGGTAAGACCCGACAGTGGATTGTTCTGGTCCATG
(C14>A)(C17>T)(A26>C)(T36>C)	GACCGCCGGGGCCAAGTGCCGAAAGCCGACGCTTGCGGGTCAGACCCGACAGCGGGTTGTTCTGGTCCATG
(G11>A)(C17>T)(A26>C)(T36>C)	GACCGCCGGGACCCAGTGCCGAAAGCCGACGCTTGCGGGTCAGACCCGACAGCGGGTTGTTCTGGTCCATG
(G8>A)(C17>T)(A26>C)(T36>C)	GACCGCCAGGGCCCAGTGCCGAAAGCCGACGCTTGCGGGTCAGACCCGACAGCGGGTTGTTCTGGTCCATG
(G5>A)(C17>T)(A26>C)(T36>C)	GACCACCGGGGCCCAGTGCCGAAAGCCGACGCTTGCGGGTCAGACCCGACAGCGGGTTGTTCTGGTCCATG
(C14>A)(C17>T)(A23>C)(T36>C)	GACCGCCGGGGCCAAGTGCCGACAGACGACGCTTGCGGGTCAGACCCGACAGCGGGTTGTTCTGGTCCATG
(G11>A)(C17>T)(A23>C)(T36>C)	GACCGCCGGGACCCAGTGCCGACAGACGACGCTTGCGGGTCAGACCCGACAGCGGGTTGTTCTGGTCCATG
(G8>A)(C17>T)(A23>C)(T36>C)	GACCGCCAGGGCCCAGTGCCGACAGACGACGCTTGCGGGTCAGACCCGACAGCGGGTTGTTCTGGTCCATG
(G5>A)(C17>T)(A23>C)(T36>C)	GACCACCGGGGCCCAGTGCCGACAGACGACGCTTGCGGGTCAGACCCGACAGCGGGTTGTTCTGGTCCATG
(C14>A)(C17>T)(C20>A)(T36>C)	GACCGCCGGGGCCAAGTGCAGAAAGACGACGCTTGCGGGTCAGACCCGACAGCGGGTTGTTCTGGTCCATG
(G11>A)(C17>T)(C20>A)(T36>C)	GACCGCCGGGACCCAGTGCAGAAAGACGACGCTTGCGGGTCAGACCCGACAGCGGGTTGTTCTGGTCCATG
(G8>A)(C17>T)(C20>A)(T36>C)	GACCGCCAGGGCCCAGTGCAGAAAGACGACGCTTGCGGGTCAGACCCGACAGCGGGTTGTTCTGGTCCATG
(G5>A)(C17>T)(C20>A)(T36>C)	GACCACCGGGGCCCAGTGCAGAAAGACGACGCTTGCGGGTCAGACCCGACAGCGGGTTGTTCTGGTCCATG
**Related to Figure 6 (coordinates from oligonucleotide 5’)**	
(C20>G)(T36>C)	GACCGCCGGGGCCCAGCGCGGAAAGACGACGCTTGCGGGTCAGACCCGACAGCGGGTTGTTCTGGTCCATG
(C20>G)(G21>C)(A22>T)(T36>C)	GACCGCCGGGGCCCAGCGCGCTAAGACGACGCTTGCGGGTCAGACCCGACAGCGGGTTGTTCTGGTCCATG
(C20>A)(G21>C)(A22>T)(T36>C)	GACCGCCGGGGCCCAGCGCACTAAGACGACGCTTGCGGGTCAGACCCGACAGCGGGTTGTTCTGGTCCATG
(C20>A)(G21>C)(A22>T)(A23>T)(T36>C)	GACCGCCGGGGCCCAGCGCACTTAGACGACGCTTGCGGGTCAGACCCGACAGCGGGTTGTTCTGGTCCATG
(C20>A)(G21>C)(A22>T)(A23>C)(T36>C)	GACCGCCGGGGCCCAGCGCACTCAGACGACGCTTGCGGGTCAGACCCGACAGCGGGTTGTTCTGGTCCATG
(C20>G)(G21>C)(A22>T)(A23>T)(T36>C)	GACCGCCGGGGCCCAGCGCGCTTAGACGACGCTTGCGGGTCAGACCCGACAGCGGGTTGTTCTGGTCCATG

(C20>G)(G21>C)(A22>T)(A23>C)(T36>C)	GACCGCCGGGGCCCAGCGCGCTCAGACGACGCTTGCGGGTCAGACCCGACAGCGGGTTGTTCTGGTCCATG
(T36>C)	GACCGCCGGGGCCCAGCGCCGAAAGACGACGCTTGCGGGTCAGACCCGACAGCGGGTTGTTCTGGTCCATG
(A26>T)(T36>C)	GACCGCCGGGGCCCAGCGCCGAAAGTCGACGCTTGCGGGTCAGACCCGACAGCGGGTTGTTCTGGTCCATG
(A26>T)(G28>T)(T36>C)	GACCGCCGGGGCCCAGCGCCGAAAGTCTACGCTTGCGGGTCAGACCCGACAGCGGGTTGTTCTGGTCCATG
(A26>T)(G28>T)(A29>T)(T36>C)	GACCGCCGGGGCCCAGCGCCGAAAGTCTTCGCTTGCGGGTCAGACCCGACAGCGGGTTGTTCTGGTCCATG
(A26>T)(G28>T)(A29>T)(G31>T)(T36>C)	GACCGCCGGGGCCCAGCGCCGAAAGTCTTCTCTTGCGGGTCAGACCCGACAGCGGGTTGTTCTGGTCCATG
(A26>T)(G28>T)(A29>T)(T36>C)	GACCGCCGGGGCCCAGCGCCGAAAGTCTTCGCTTGCGGGTCAGACCCGACAGCGGGTTGTTCTGGTCCATG
(A23>G)(A26>T)(T36>C)	GACCGCCGGGGCCCAGCGCCGAGAGTCGACGCTTGCGGGTCAGACCCGACAGCGGGTTGTTCTGGTCCATG
(A26>T)(A29>T)(T36>C)	GACCGCCGGGGCCCAGCGCCGAAAGTCGTCGCTTGCGGGTCAGACCCGACAGCGGGTTGTTCTGGTCCATG
(A26>T)(A29>T)(C32>T)(T36>C)	GACCGCCGGGGCCCAGCGCCGAAAGTCGTCGTTTGCGGGTCAGACCCGACAGCGGGTTGTTCTGGTCCATG
(A23>G)(A26>T)(A29>T)(T36>C)	GACCGCCGGGGCCCAGCGCCGAGAGTCGTCGCTTGCGGGTCAGACCCGACAGCGGGTTGTTCTGGTCCATG
(C20>T)(A23>G)(A26>T)(A29>T)(T36>C)	GACCGCCGGGGCCCAGCGCTGAGAGTCGTCGCTTGCGGGTCAGACCCGACAGCGGGTTGTTCTGGTCCATG
(C20>T)(A23>G)(A26>T)(A29>T)(C32>T)(T36>C)	GACCGCCGGGGCCCAGCGCTGAGAGTCGTCGTTTGCGGGTCAGACCCGACAGCGGGTTGTTCTGGTCCATG
